# High-precision fault inception time detection in active distribution networks using Euclidean distance and Gaussian filtering

**DOI:** 10.1371/journal.pone.0359598

**Published:** 2026-10-09

**Authors:** Haiou Cao, Yihua Meng, Chenbin Zhou, Lichen Deng, Jiayan Yu

**Affiliations:** 1 State Grid Jiangsu Electric Power Company, Nanjing, Jiangsu, China; 2 State Grid Suzhou Power Supply Company, Suzhou, Jiangsu, China; BVRIT Hyderabad College of Engineering For Women, INDIA

## Abstract

Fault signals in power systems often contain high-order harmonics and attenuated oscillations. which pose challenges for traditional fault time detection methods in accurately identifying the fault inception instant. To address this issue, this paper proposes an accurate fault time detection method based on Euclidean distance and Gaussian filter. Based on the phase voltage or current signal of the fault line, the improved high-order Fourier algorithm with attenuation factor is used to fit the curve of the fault phase signal data. The fault signal is compared with the imaginary non-fault signal by using Euclidean distance, and the fitting error is filtered by a Gaussian filtering algorithm to detect the fault segment. Finally, the fault is detected by pixel column, and the fault time is obtained by corresponding the pixel column and time information. The MATLAB/Simulink simulation results and the field test results show that the method can accurately detect the fault time under different harmonics, damped oscillations, transition resistance, short-circuit fault types and sampling rates, and the detection error is basically controlled within 0.1 ms, which achieves high detection accuracy. Combining the advantages of both computational efficiency and detection accuracy, the proposed method demonstrates superior practicality and cost-effectiveness in active distribution networks when compared to existing technologies.

## 1. Introduction

The extensive integration of distributed generation has transformed the structure of distribution networks from traditional radial networks to multi-source networks [1–3]. As a result, the fault characteristics of distribution systems have become more complex, with more pronounced issues such as harmonics and damped oscillations. This poses new challenges for real-time fault detection and protection in active distribution networks [4–6]. In the application of relay protection in active distribution networks, differential protection and fault location technologies primarily rely on the synchronization of electrical quantities at both ends of the line [7–9]. Currently, traditional fault self-synchronization algorithms achieve low-cost data synchronization by aligning the detected fault instants on the power supply side and the load side [10,11]. However, when there is a significant discrepancy in fault detection times between the two ends, this algorithm may introduce substantial measurement errors, potentially causing protection devices to fail to operate promptly [12–14].

The key to achieving reliable relay protection in active distribution networks lies in the accurate determination of the fault inception instant [15]. As the primary stage of relay protection systems, the more reliable the fault detection module, the faster the protection devices can respond to faults [16]. The fault detection module continuously monitors electrical parameter variations and triggers protective actions upon detecting abrupt changes exceeding predefined thresholds [17]. Precise determination of the fault inception instant is critical for subsequent protection operations and fault clearance [18]. In active distribution networks with distributed generation, the complex and variable nature of fault characteristics makes it challenging for traditional fixed-setting initiation criteria to reliably distinguish between fault and non-fault conditions [19–22]. Therefore, precise detection of the fault inception instant is of paramount importance for enhancing the reliability, selectivity, speed, and sensitivity of relay protection in active distribution networks [23].

In recent years, several researchers have investigated methods for fault moment detection. Liu et al. [23] utilized the three-phase voltage data collected by Phasor Measurement Unit (PMU) at each node to construct a data source matrix. By analyzing the normal distribution curves of the column data in the time window matrix, the fault moment was determined. However, the moving time window is influenced by historical data, resulting in relatively high detection errors. Dong et al. [24] used the parameters of the phase current transient component using the Prony algorithm, calculated the zero-crossing points of the phase current transient component fitting function, and identified the actual fault moment. This method demonstrates good detection performance, but its accuracy is affected by the data window length and sampling frequency. Zhang et al. [25] combined the phase current transient component with mathematical morphology, processing the phase current signal using an improved multi-resolution morphological gradient operator. When the sampling rate decreases, the detection error on the distributed generation side may exceed one sampling interval. Li et al. [26] constructed a Hankel matrix from discrete fault signals and employed singular value decomposition to separate the signals. The moment of fault occurrence is determined by calculating the modulus maxima of the detail components. However, the number of columns in the Hankel matrix requires manual selection and lacks an adaptive mechanism, which may compromise the results if chosen inappropriately. Yan et al. [27] employed the Prony algorithm to fit and extrapolate the original signal, generating a hypothetical non-fault signal. By performing pixel-by-pixel column comparison between the original and fitted signals, the method achieves pixel-level accuracy in fault timing detection. However, the approach does not address the detection of damped oscillatory components and higher-order harmonics commonly present in power systems. Yao et al. [28] developed an active high-impedance fault detection method for resonant grounding distribution networks based on fault-feature enhancement, achieving reliable detection of 75kΩ transition impedance faults under 15dB noise conditions. Najafzadeh et al. [29] proposed a fault detection, classification, and localization method for transmission lines using fuzzy logic thresholding, WHO-optimized DT/RF, and ANFIS. It achieved 100% accuracy and <153.6m localization error in simulations. However, it relies solely on frequency signals for features and the WHO algorithm is sensitive to parameter settings, affecting generalization stability. Li et al. [30] designed an AD-TFM unit with learnable wavelet parameters embedded in LSTM and attention mechanism for incipient fault timing detection. The method adaptively extracts weak fault features via learnable time-frequency transforms and augments scarce samples through phase-switching and time-sliding strategies. However, it is limited to fault detection without classification or localization, and its RNN-based structure faces computational efficiency issues with long sequences or large-scale networks.

Despite the variety of existing fault detection methods, accurately identifying fault occurrence times under complex conditions—such as harmonic interference, damped oscillations, and low sampling rates—remains a persistent challenge. To address this issue, this paper proposes a novel fault-time detection method based on Euclidean distance and Gaussian filtering, specifically designed for active distribution networks. The approach begins by applying an enhanced high-order Fourier algorithm with a damping factor to achieve high-fidelity fitting of fault current signals containing harmonics and oscillatory components. Following this, a hybrid image-based strategy combining Euclidean distance and Gaussian filtering is used to translate waveform similarities into pixel-level comparisons, allowing precise segmentation of the fault interval. Finally, pixel-wise column analysis within the identified segment enables precise localization of the fault inception time. Simulation results confirm that the method maintains detection errors below 0.1 ms under diverse scenarios including harmonic distortion, oscillatory decay, varying transition resistances, multiple fault types, and reduced sampling rates, outperforming existing techniques. Field data validation has confirmed the practicality of the proposed method. This study enhances the accuracy and adaptability of fault diagnosis in active distribution networks, offering a computationally efficient and highly practical solution for real-world grid monitoring and automation.

## 2. High-order Fourier algorithm with attenuation factor

Accurate identification of fault occurrence time in active distribution networks is crucial for ensuring rapid and reliable protection responses. However, the increasing integration of distributed generation has led to more complex fault characteristics, including pronounced harmonics and damped oscillatory components. These non-stationary signal features pose significant challenges to conventional detection methods, which often fail to represent transient behaviors accurately. High-fidelity signal fitting thus becomes essential, as it forms the foundation for precise fault analysis and subsequent timing detection.

Fourier function fitting is a mathematical method based on Fourier series or Fourier transform, used to approximate or fit complex signals, probability density functions, or other data distributions. It has wide applications in fields such as signal processing, image analysis, and probability modeling [31]. The high-order Fourier algorithm significantly enhances the fitting of complex fault waveforms in power systems by incorporating higher-order harmonic components. This enables precise reconstruction of signals containing harmonics and oscillatory transients, which are often challenging for conventional methods. The high-order Fourier fitting can be summarized as follows:


$$\begin{array}{c} \left\{ \begin{array}{c} @l@f\left(t\right)=a_{\text{0}}+\sum_{i=1}^{n}\left[a_{i}cos\left(\frac{2\pi it}{T})+b_{i}sin(\frac{2\pi it}{T}\right)\right]\\ a_{\text{0}}=\frac{\text{1}}{T}\int_{\text{0}}^{T}f\left(t\right)\text{d}t\\ a_{i}=\frac{\text{2}}{T}\int_{\text{0}}^{T}f\left(t\right)cos\left(\frac{2\pi it}{T}\right)\text{d}t\\ b_{i}=\frac{\text{2}}{T}\int_{\text{0}}^{T}f\left(t\right)sin\left(\frac{2\pi it}{T}\right)\text{d}t \end{array}\right. \end{array}$$ (1)

where f(t) is the fitting expression obtained for approximating the raw signal.; $a_{\text{0}}$ is the direct current offset coefficient obtained from fitting; n is the maximum fitting harmonic order; T is the fitted signal period; $a_{i}$ and $b_{i}$ are the amplitude coefficients of the i-th order cosine and sine components, respectively.

Damped oscillatory components may occasionally occur in power systems, primarily caused by transient processes such as faults, switching operations, load variations, resonances, and external disturbances. These transient processes cause the inductive and capacitive elements in the system to form oscillatory circuits, generating damped oscillatory signals. However, conventional high-order Fourier algorithms fail to account for damped oscillatory components. To address this limitation, a damping factor is incorporated into (1), with the complete mathematical formulation given in (2).


$$\begin{array}{c} f\left(t\right)=a_{\text{0}}+\sum_{i=1}^{n}\left[a_{i}cos\left(\frac{2\pi it}{T})+b_{i}sin(\frac{2\pi it}{T}\right)\right]\cdot e^{\left(c_{i}t\right)} \end{array}$$ (2)

where $c_{i}$ is the damping factor of the i-th order cosine and sine frequency components, its appropriate value is estimated through the nonlinear least squares method.

The high-order Fourier algorithm, when incorporating a damping factor, can achieve high-precision fitting for signals containing harmonics and damped oscillations. $I_{a}$ is the fault phase current. The limitation of the conventional Fourier fitting algorithm is shown in Fig 1, where the signal is simply decomposed into a superposition of trigonometric functions, failing to accurately represent damped oscillations and higher-order harmonics present in the original waveform. Fig 2 presents the improved Fourier fitting algorithm proposed in this study, where the fault signal and the fitted extended signal show nearly perfect alignment. Even with the inclusion of damped oscillatory components and higher-order harmonics, the algorithm maintains precise fitting accuracy for both the damped oscillations and high-frequency harmonic components in the signal. By incorporating a damping factor, the enhanced method effectively addresses the limitations of traditional approaches in accurately modeling complex fault waveforms that contain both harmonics and damped oscillations, thereby establishing a solid foundation for subsequent comparative analysis between the original and fitted extended signals. The raw data used to generate Figs 1, 2, and 7 are provided in S1 Data.

**Fig 1:**
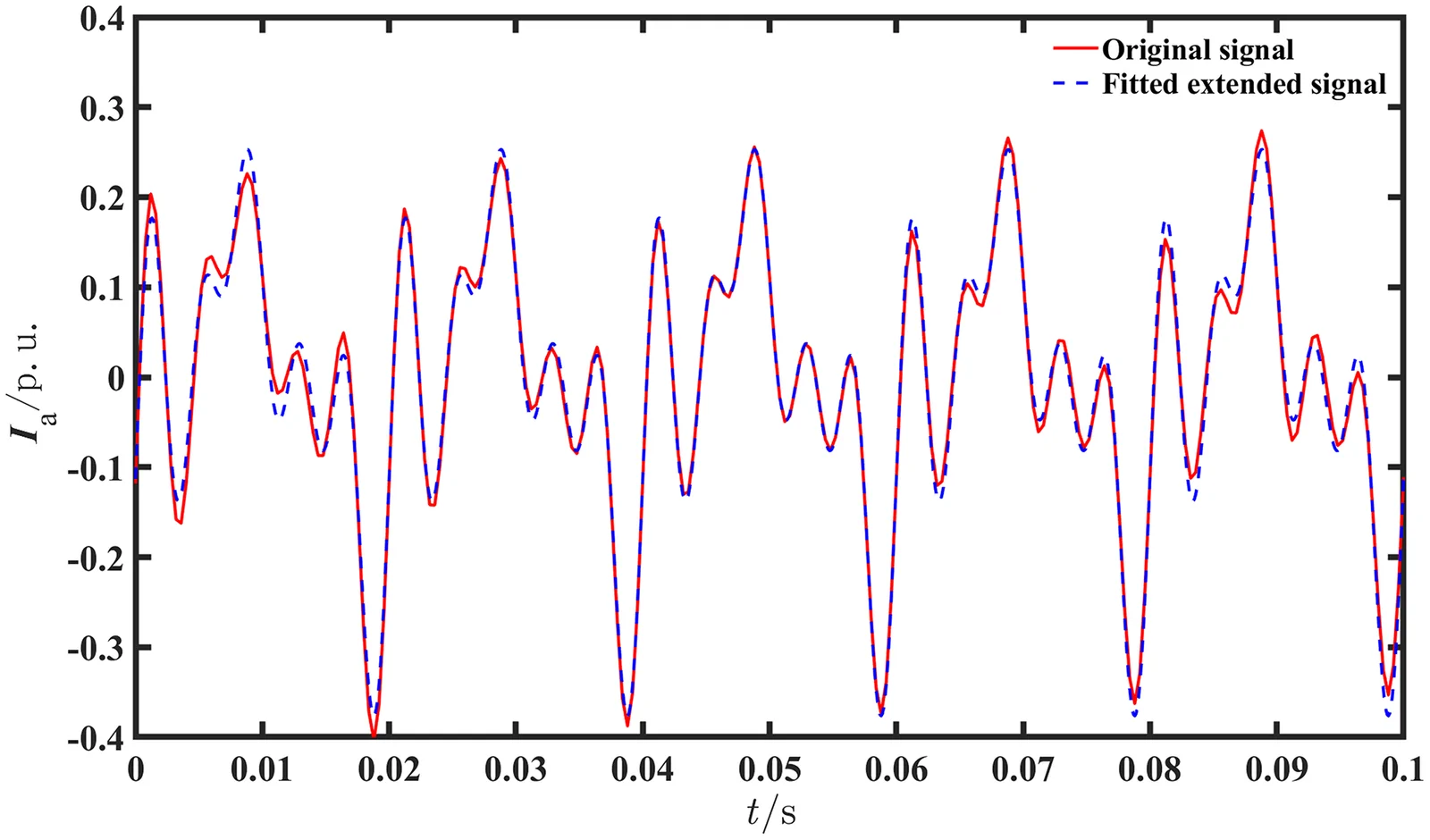
The fitting performance of the conventional Fourier algorithm.

**Fig 2:**
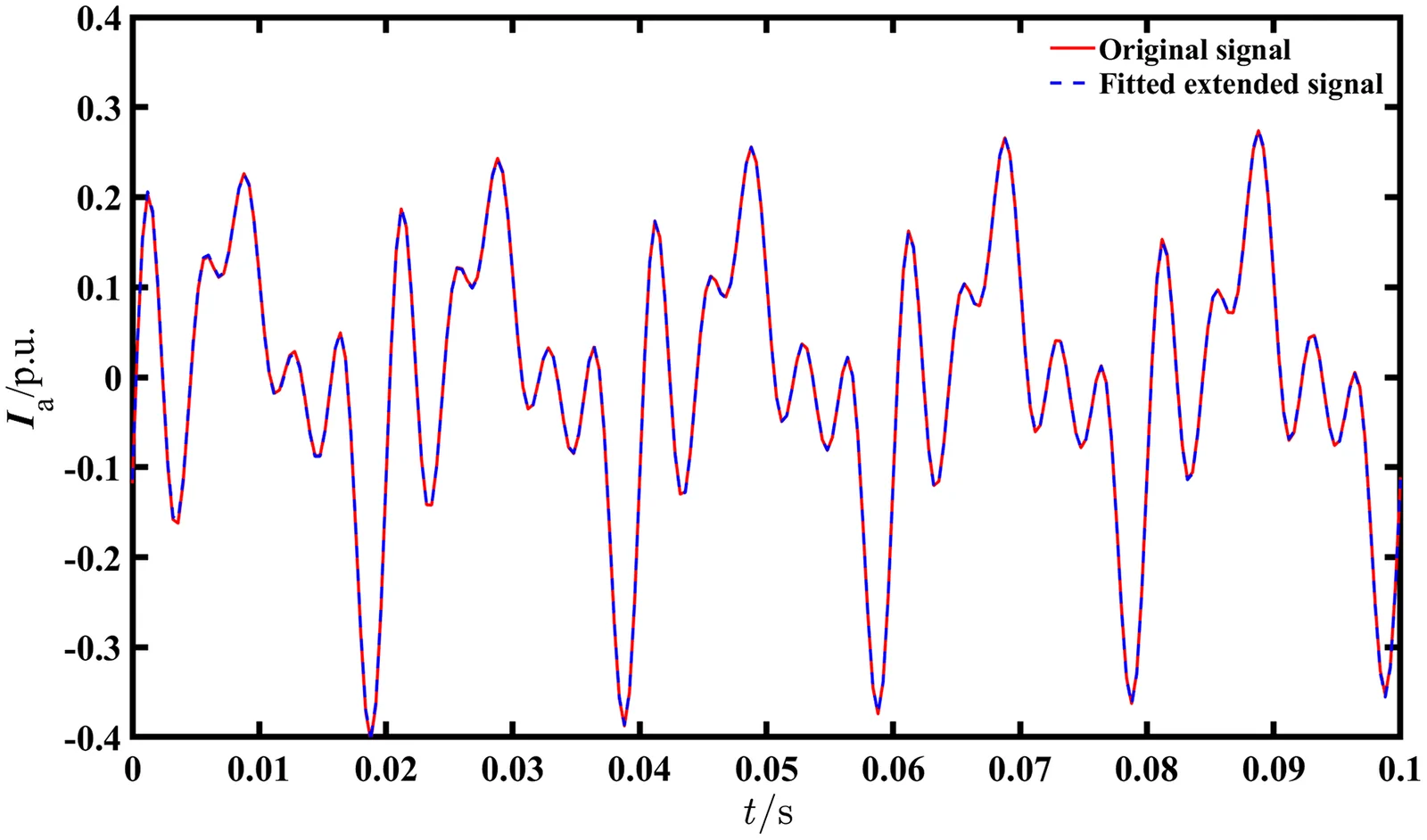
The fitting performance of the improved Fourier algorithm.

## 3. Fault instant identification through image comparison

### 3.1 Euclidean distance for image comparison

Following the high-fidelity fitting and extension of complex fault signals using the enhanced Fourier algorithm, the accurate extraction of fault characteristics becomes essential for precise timing identification. While the fitted signal provides a reference waveform, the critical challenge remains how to effectively quantify the discrepancy between the original fault signal and its fitted extension, especially under noisy and complex operating conditions.

The Euclidean distance algorithm provides a quantitative and computationally efficient approach to measuring dissimilarities between the original fault signal and its fitted extension [32,33]. By transforming the signals into vector space and evaluating pixel-wise deviations, it effectively captures waveform anomalies caused by faults. This method enhances detection sensitivity and facilitates accurate identification of fault segments, even under noisy conditions or in the presence of harmonic distortions. In this paper, image pixels are converted into one-dimensional vectors, and the Euclidean distance between images is computed to quantitatively measure their degree of difference. The proposed method implements image comparison based on Euclidean distance through the following steps (Taking the current signal as an example): First, the phase current measurements are extracted from the fault-phase current by employing high-accuracy current transformers, ensuring minimal amplitude and phase errors during signal acquisition. Next, uniform data window segmentation is applied according to the period of the fault-phase current. In parallel, two images are read and adjusted to the same duration to ensure dimensional consistency. Subsequently, grayscale conversion techniques from the field of image processing are applied to convert RGB images into grayscale images in order to eliminate the interference of color information with the difference metric, as the three RGB channels may introduce unnecessary noise that compromises the accuracy and robustness of image comparison based on algorithms such as Euclidean distance. The grayscale conversion formula is as follows:


$$\begin{array}{c} I_{\text{gray}}=0.2989*R+0.5870*G+0.1140*B \end{array}$$ (3)

where $I_{\text{gray}}$ is gray value of the image,R is red channel intensity,G is green channel intensity,B is blue channel intensity.

Subsequently, the algorithm reshapes the two grayscale images into one-dimensional vectors to facilitate Euclidean distance measurement. The vectorization process can be expressed as:


$$\begin{array}{c} 𝐕=I_{\text{gray}}(:) \end{array}$$ (4)

Finally, the Euclidean distance metric is employed to measure the dissimilarity between the two images, providing a quantitative assessment of their differences. The mathematical formulation is as follows:


$$\begin{array}{c} D={\|{𝐕}_{\text{1}}-{𝐕}_{\text{2}}\|}_{\text{2}}=\sqrt{\sum_{i=1}^{n}({𝐕}_{\text{1}}(i)-{𝐕}_{\text{2}}(i){)}^{\text{2}}} \end{array}$$ (5)

where n is dimension of a vector, ${𝐕}_{\text{1}}\left(i\right)$ and ${𝐕}_{\text{2}}\left(i\right)$ are the i-th elements of vector ${𝐕}_{\text{1}}$ and vector ${𝐕}_{\text{2}}$, respectively.

Assume there are two tiny 2x2 images. The pixel matrix of image A representing the raw signal and the pixel matrix of Image B representing the fitted signal are shown below.


$$\begin{array}{c} A=\left[\begin{array}{cc} @cc@a_{\text{1}} & a_{\text{2}}\\ a_{\text{3}} & a_{\text{4}} \end{array}\right] \end{array}$$ (6)

$$\begin{array}{c} B=\left[\begin{array}{cc} @cc@b_{\text{1}} & b_{\text{2}}\\ b_{\text{3}} & b_{\text{4}} \end{array}\right] \end{array}$$ (7)

where $a_{\text{1}}$, $a_{\text{2}}$, $a_{\text{3}}$ and $a_{\text{4}}$ represent the grayscale values of individual pixels in image A. $b_{\text{1}}$, $b_{\text{2}}$, $b_{\text{3}}$ and $b_{\text{4}}$ represent the grayscale values of individual pixels in image B.

Convert the two N-dimensional vectors into one-dimensional vectors in the form of $\left[\begin{array}{cccc} @cccc@a_{\text{1}} & a_{\text{2}} & a_{\text{3}} & a_{\text{4}} \end{array}\right]$ and $\left[\begin{array}{cccc} @cccc@b_{\text{1}} & b_{\text{2}} & b_{\text{3}} & b_{\text{4}} \end{array}\right]$. The Euclidean distance D between two N-dimensional vectors $A=\left[\begin{array}{cccc} @cccc@a_{\text{1}} & a_{\text{2}} & a_{\text{3}} & a_{\text{4}} \end{array}\right]$ and$B=\left[\begin{array}{cccc} @cccc@b_{\text{1}} & b_{\text{2}} & b_{\text{3}} & b_{\text{4}} \end{array}\right]$ is calculated by the formula:


$$\begin{array}{c} D=\sqrt{\begin{array}{c} (a_{\text{1}}-b_{\text{1}}{)}^{\text{2}}+(a_{\text{2}}-b_{\text{2}}{)}^{\text{2}}+\\ (a_{\text{3}}-b_{\text{3}}{)}^{\text{2}}+(a_{\text{4}}-b_{\text{4}}{)}^{\text{2}} \end{array}} \end{array}$$ (8)

Thus, we obtain the Euclidean distance between the corresponding segments of the original signal and the fitted extended signal, laying the foundation for determining the segment where the fault occurrence time is located.

As shown in Fig 3, A fitted extended signal is obtained by fitting and extending the pre-fault signal (one cycle of non-fault data) to match the length of the raw signal. The raw signal exhibits significant oscillations after a fault occurs, which contrasts sharply with its previously stable non-fault state. The fitted extended signal extends according to the pre-fault pattern and remains relatively stable. Comparing these two signals in the same graph can vividly demonstrate the signal changes caused by the fault and help determine the time when the fault occurs. The raw data used to generate Fig 3 is provided in S2 Data.

**Fig 3:**
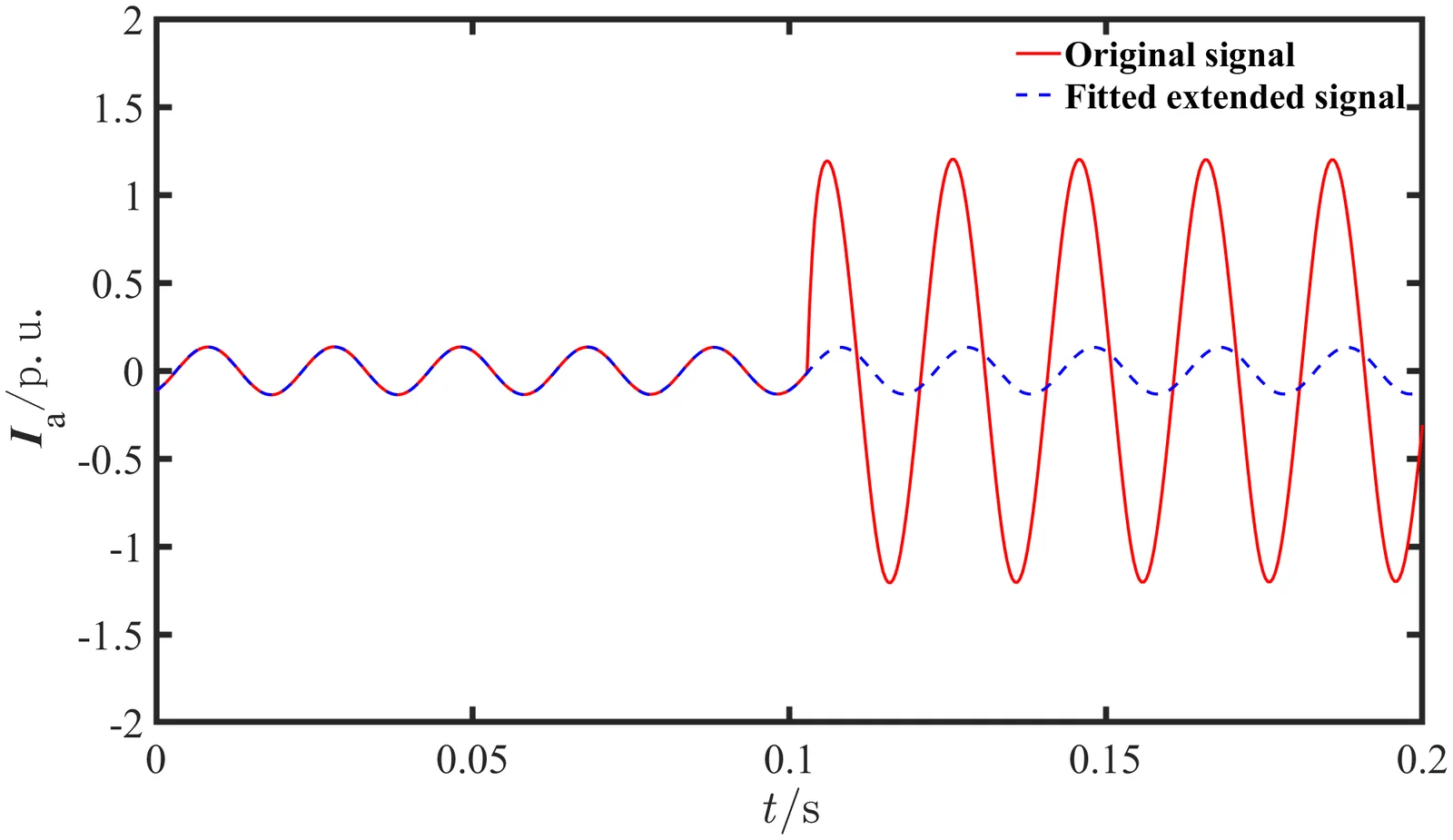
Comparison between the raw signal and the fitted extended signal without harmonics and attenuation factors.

The raw signal and the fitted extended signal in Fig 3 are compared periodically, with each cycle having a duration of 0.02 s. The Euclidean distance of each segment was calculated using (5), with the results shown in Fig 4, which illustrates the variation trend of Euclidean distances across different segments. The horizontal axis represents the Segment, numbered from 1 to 10, whereas the vertical axis represents the Euclidean distance values, which are dimensionless quantities computed from the grayscale vectors of the segmented signal images. In Segments 1–5, the Euclidean distances are relatively small, with all values falling below the predefined threshold. This stage corresponds to the normal operation phase. At this time, the differences between the fault signal and the fitted extended signal are extremely small, resulting in low Euclidean distance values. In Segment 6, The Euclidean distance exceeds the threshold for the first time. This is the moment when the fault initiates. The sudden increase in the difference between the fault signal and the fitted extended signal is reflected in the sharp rise of the Euclidean distance. For Segments 7–10, the Euclidean distances remain at a relatively high level and are relatively stable, indicating that after the fault occurs, a persistent large difference exists between the two signals. By observing the abrupt change in the Euclidean distance, the segment where the fault moment occurs can be determined, providing important evidence for fault detection and analysis.

**Fig 4:**
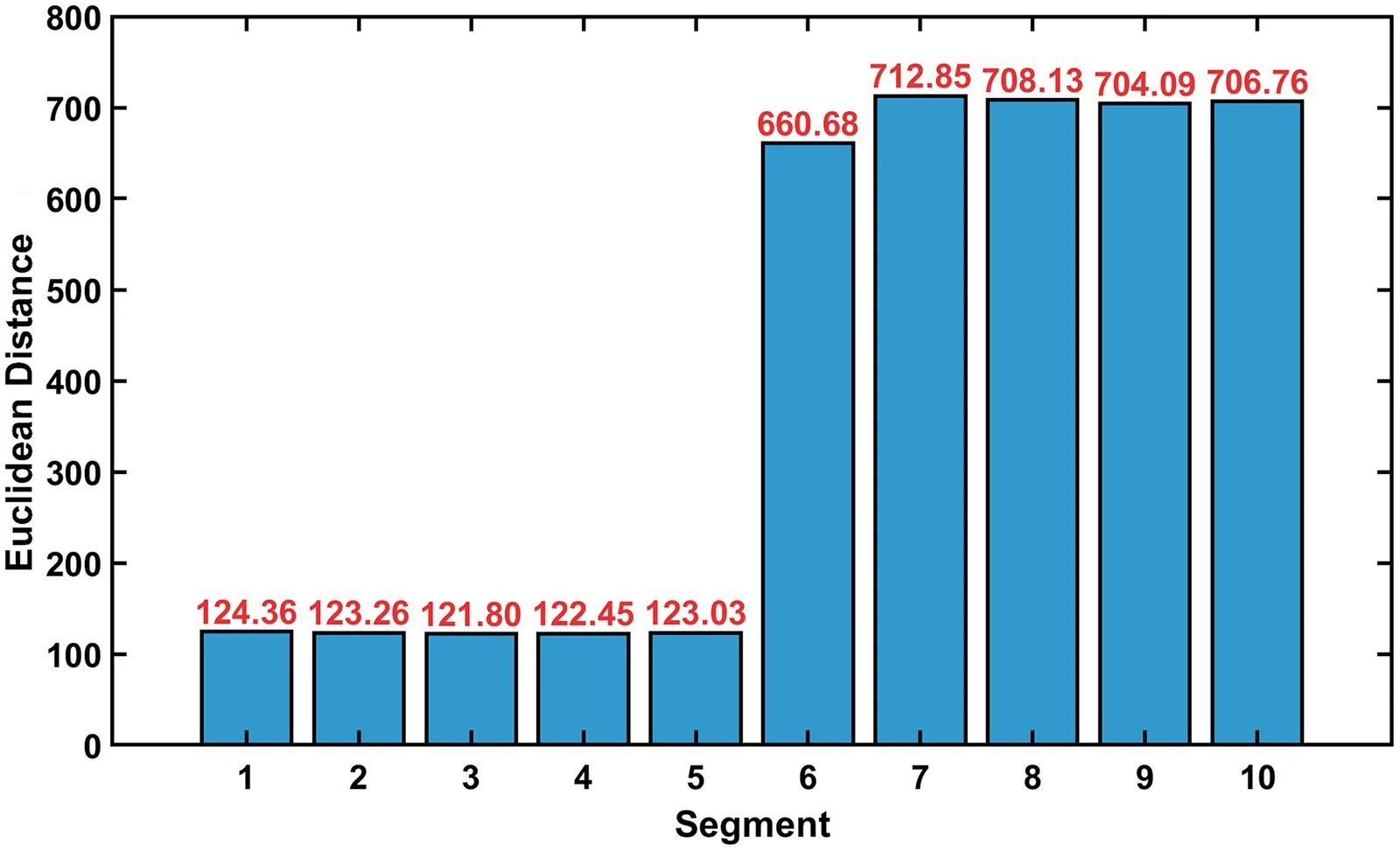
Euclidean distance for each segment.

Based on the aforementioned results, a criterion for fault segment discrimination has been designed. A faulty segment is identified by comparing the Euclidean distance of each individual segment with the product of the maximum Euclidean distance among all segments and the localization coefficient. The mathematical criterion is formally expressed as follows:


$$\begin{array}{c} D^{k}\geq \varepsilon •max\left[D^{\text{1}},D^{\text{2}},\cdots ,D^{n_{e}}\right] \end{array}$$ (9)

where $D^{k}$ is the Euclidean distance calculated for the k-th segment of the two signal images,ε is the localization coefficient, $n_{e}$ is the number of raw signal segment and fitted extended signal segment, $D^{\text{1}},D^{\text{2}},\cdots ,D^{n_{\text{e}}}$ are the Euclidean distances calculated between segment 1 to segment $n_{e}$ of these two signals.

Empirically, ε is set within 0.2–0.3 based on statistical analysis of 200 fault cases under varying harmonics, damped oscillations, and transition resistances. For non-fault segments, the normalized Euclidean distance $D_{k}$ has a mean of 0.087, with 99.7% of samples below 0.18. For fault-inception segments, the mean is 0.472, with 99.7% of samples above 0.21. Consequently, setting ε within 0.2–0.3 limits the false positive rate for non-fault segments to below 0.5% and the false negative rate for fault segments to below 0.1%. Sensitivity analysis further confirms that within this range, the detection error remains stable at 0.085 ms with a 100% fault segment localization success rate. By quantifying signal differences through Euclidean distance, this method resolves the challenge of automatically and accurately pinpointing the initiation zone of faults. The technique converts signals into image vectors and captures fault-induced abrupt changes by computing their spatial distance, thereby overcoming the insufficient sensitivity of traditional fixed-threshold methods under noise and harmonic interference, and ultimately achieving intelligent identification of fault segments.

### 3.2 Gaussian filtering for error suppression and noise smoothing

Building upon the effective identification of the fault time segment achieved in the previous section, further refinement is required to accurately determine the precise fault inception time. To address this need, an image comparison method based on Gaussian filtering is proposed. This approach effectively eliminates minor fitting errors and suppresses noise interference through signal smoothing, thereby significantly enhancing the accuracy and robustness of fault timing detection [34]. The detailed implementation method is as follows. First, load the images of the normal signal and fitted extended signal corresponding to the fault segment, verifying consistent image dimensions. Resize mismatched images to ensure uniform sizing, which is essential for proper image alignment and differential analysis in subsequent steps. Next, apply Gaussian filtering to mitigate minor fitting errors.

When employing higher-order Fourier algorithms for fault signal fitting and extension, minor errors emerge at function peaks. These minor errors adversely impact the precision of subsequent fault timing detection. To obtain accurate fault timing results, it is essential to eliminate these minor errors. Gaussian filtering smooths signals by essentially performing a convolution operation on the image using a Gaussian kernel generated from the Gaussian function, thereby eliminating scattered noise interference [35]. We employ Gaussian filtering to process minor fitting errors. The specific implementation method is as follows.

The Gaussian filter performs a weighted average on the target pixel and its neighboring pixels, with the weights determined by the Gaussian function. The Gaussian function is given by (7). The resulting 3 × 3 Gaussian kernel is shown in (8).


$$\begin{array}{c} G\left(x,y\right)=\frac{\text{1}}{2\pi \sigma^{\text{2}}}e^{-\frac{x^{\text{2}}+y^{\text{2}}}{2\sigma^{\text{2}}}} \end{array}$$ (10)

where (x,y) is the coordinates of the pixel points.σ is the standard deviation of the Gaussian function, which controls the smoothness of the filter. G(x,y) is the weight value of the Gaussian kernel at point (x,y).


$$\begin{array}{c} \left[\begin{array}{ccc} @ccc@1 & 2 & 1\\ 2 & 4 & 2\\ 1 & 2 & 1 \end{array}\right] \end{array}$$ (11)

The Gaussian filter uses a 3 × 3 kernel with a standard deviation σ=0.5. This choice is based on the observation that the high-order Fourier fitting produces minor fitting errors at the function peaks. The selected 3 × 3 kernel with σ=0.5 effectively smooths these minor errors while preserving the sharp fault-induced transient. Sensitivity analysis shows that when σ varies within 0.3–0.8, the detection error remains below 0.1 ms. When σ is too large (>1.0), the signal becomes oversmoothed, and the detection error increases to above 0.15 ms.

For each pixel, a weighted average is computed using its neighboring pixels within the defined kernel region. The weighting coefficients follow a Gaussian distribution, where pixels farther from the center receive progressively smaller weights. This weighted average then replaces the original pixel value.


$$\begin{array}{c} \begin{array}{c} \left[\begin{array}{ccc} @ccc@40 & 107 & 5\\ 198 & 226 & 223\\ 37 & 68 & 193 \end{array}\right]*\frac{\text{1}}{1*4+2*4+8}\\ \left[\begin{array}{ccc} @ccc@1 & 2 & 1\\ 2 & 8 & 2\\ 1 & 2 & 1 \end{array}\right]=\left[\begin{array}{ccc} @ccc@ & & \\ & 164 & \end{array}\right] \end{array} \end{array}$$ (12)

In Gaussian filtering, a convolution kernel scans each pixel in the image. The original center pixel value (e.g.,226) is replaced by a new value (e.g.,164), computed as the weighted average of neighboring pixel intensities determined by the Gaussian kernel. This process effectively eliminates minor fitting errors.

Finally, compute the absolute pixel-wise difference between the two filtered images to generate a difference image. Specifically, the algorithm begins at the top-left pixel located at coordinate 0,0 of the image and proceeds to traverse sequentially either by row or by column until it reaches the bottom-right pixel at coordinate W-1, H-1. Here, W represents the image width, which is the number of pixel columns, and H represents the image height, which is the number of pixel rows. For each pixel at position x, y in the image, the grayscale values from the corresponding positions in image A and image B are obtained, and the absolute difference between them is computed. This resulting absolute difference value is then assigned to the pixel at the same position x, y in the difference image C. In regions where significant discrepancies occur between the original signal and the fitted extended signal—such as image areas corresponding to the occurrence or duration of a fault—the pixel values at these corresponding positions in the difference image will be substantially greater than zero. At regions with zero pixel values, the difference image will appear blank; whereas areas with significantly non-zero pixel values will highlight the dissimilarities between the original images. This difference image visually illustrates the discrepancy distribution between the two images, providing crucial data support for subsequent fault timing localization. Pixel differences can be expressed as follows:


$$\begin{array}{c} C\left(x,y\right)=\left|A\left(x,y\right)-B\left(x,y\right)\right| \end{array}$$ (13)

where A(x,y), B(x,y) and C(x,y) are the pixel value at position (x, y) in image *A*, *B* and *C*, respectively.

After obtaining the pixel differences of the images, the algorithm will automatically annotate the differential line segments and identify the pixel column containing the first differing pixel. Figs 5 and 6 show the fault detection diagram obtained by calculating the Euclidean distance and applying Gaussian filtering to Segment 6 of the fault signal. The analysis of the figure reveals that all signal segments preceding the pixel column corresponding to the fault instant were discarded during processing, as no discernible deviation was detected between the original signal and its fitted extension. In contrast, subsequent segments exhibiting statistically significant deviations were retained for further analysis. The first detected pixel column with non-negligible divergence was algorithmically identified and annotated as the precise fault initiation instant.

**Fig 5:**
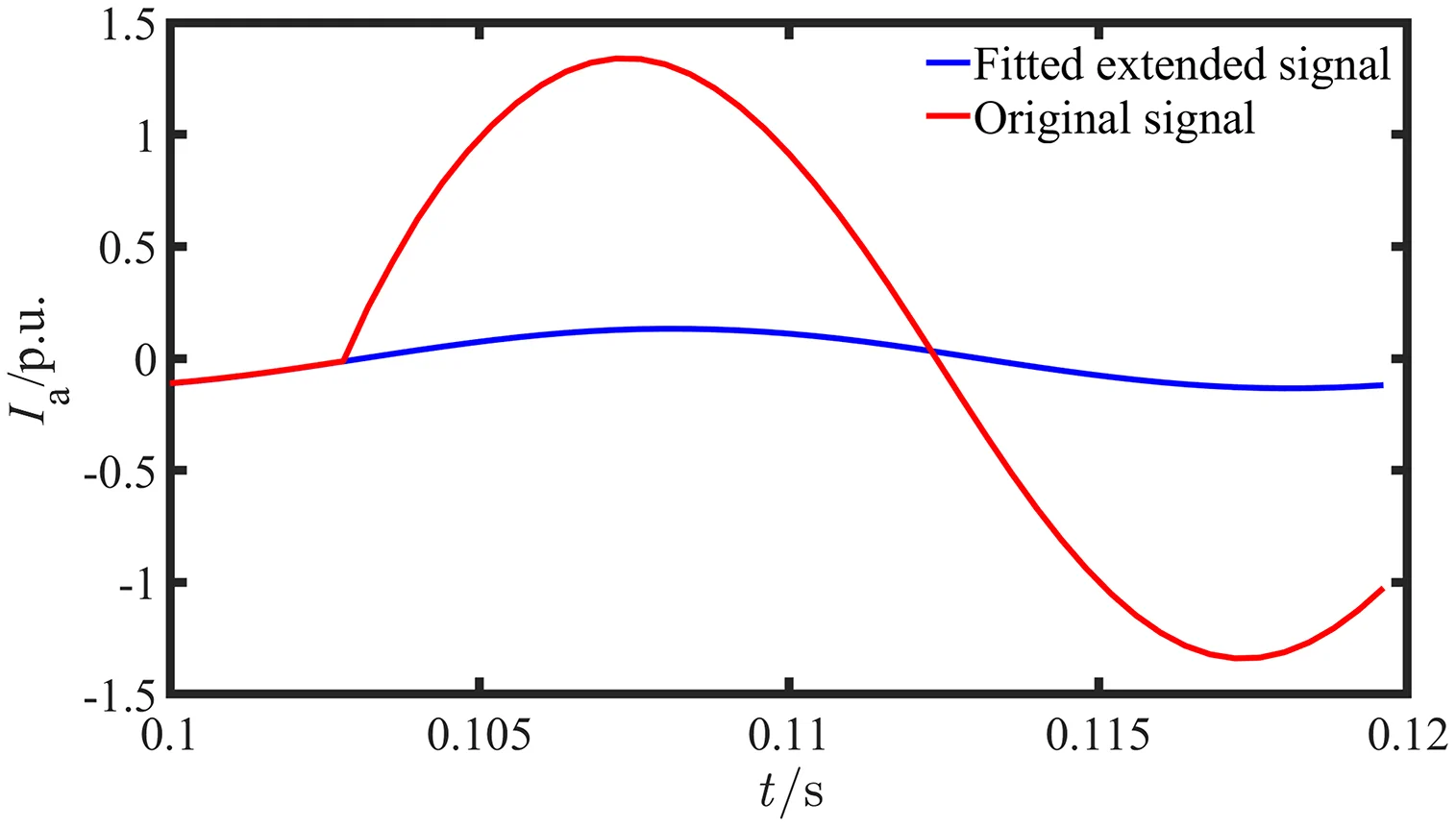
Raw data for pixel column fault detection.

**Fig 6:**
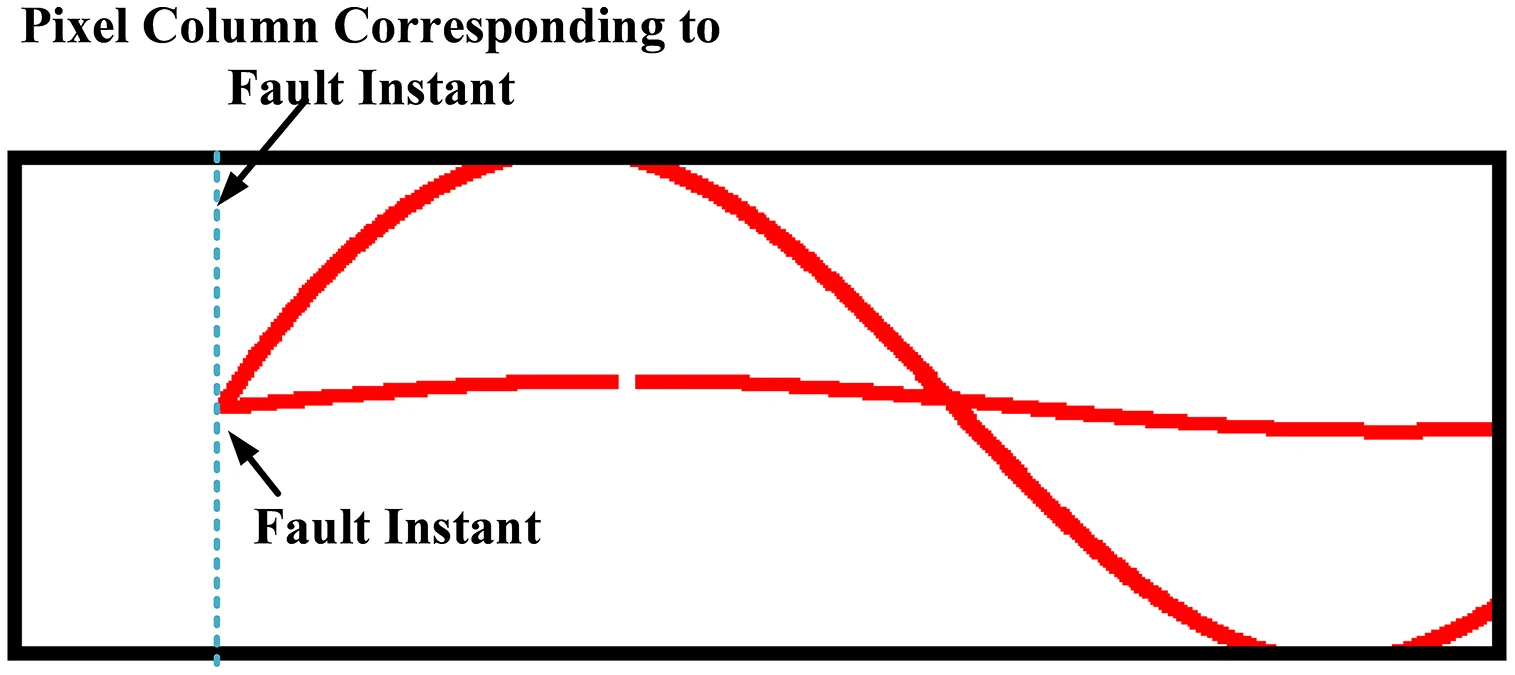
Comparative image recognition for pixel column fault detection.

By performing pixel-wise comparison between two images, differences can be localized with single-pixel precision. Based on the first differing pixels, the exact pixel column where a fault occurs can be identified. Correlating the detected pixel columns with temporal data enables precise fault timing detection, achieving an error margin of just one pixel.

Based on the signal in Fig 3, a damped oscillation signal with a damping factor of −10 is added, along with third-order harmonics and fifth-order harmonics. As seen from Fig 7, the improved high-order Fourier algorithm can correctly fit the raw signal and extend it to obtain the fitted extended signal. Fig 8 presents the initial fault stage identified using the Euclidean distance. As shown in the pixel column fault detection diagram of Fig 9, the application of Gaussian filtering eliminates scattered pixel artifacts and suppresses fitting errors, resulting in a clean post-fault signal profile. The fault timing can be precisely determined by detecting the specific pixel column where the fault initiates.

**Fig 7:**
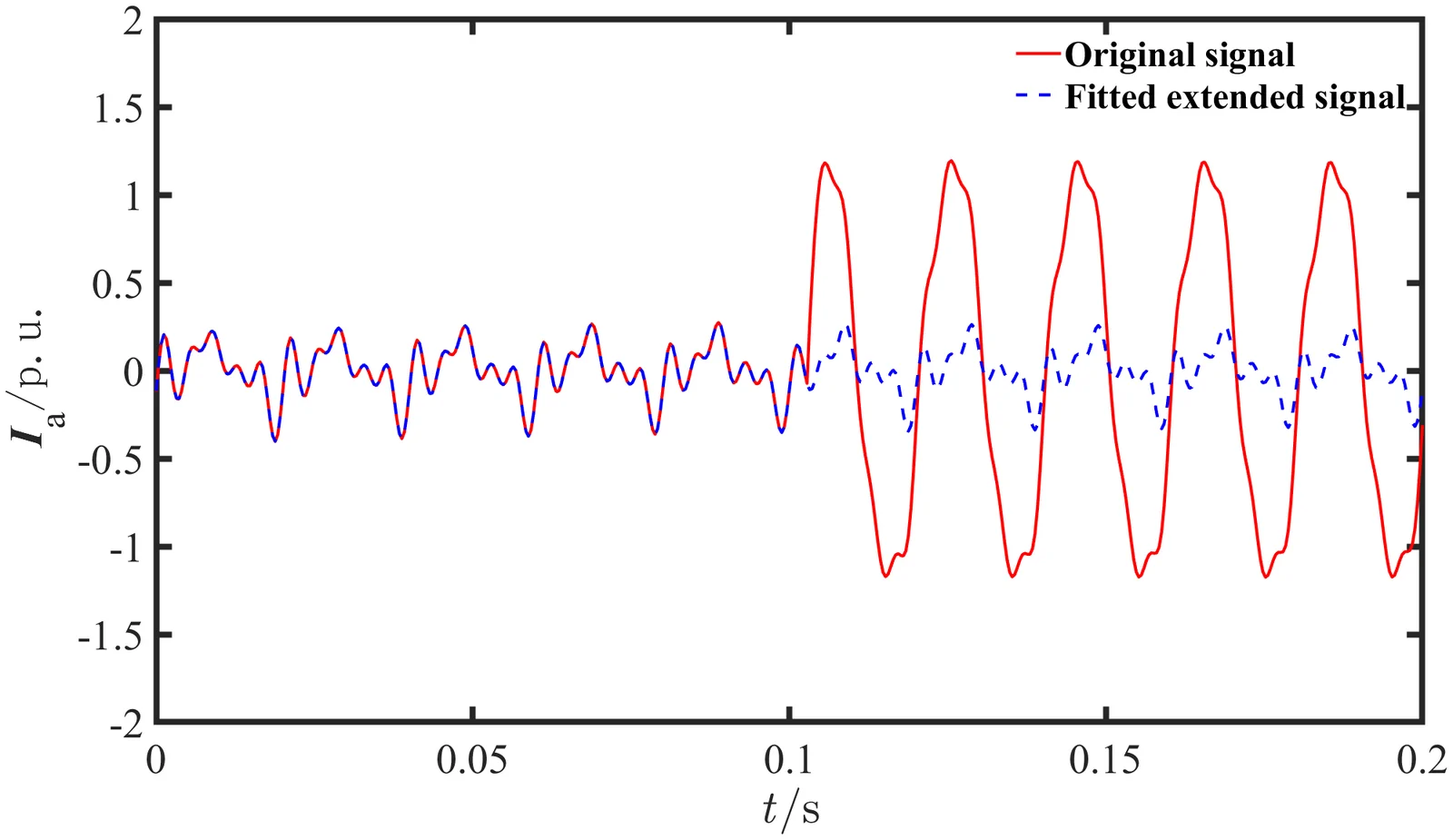
Comparison of the fault signal containing harmonics and attenuation factors with the fitted extended signal.

**Fig 8:**
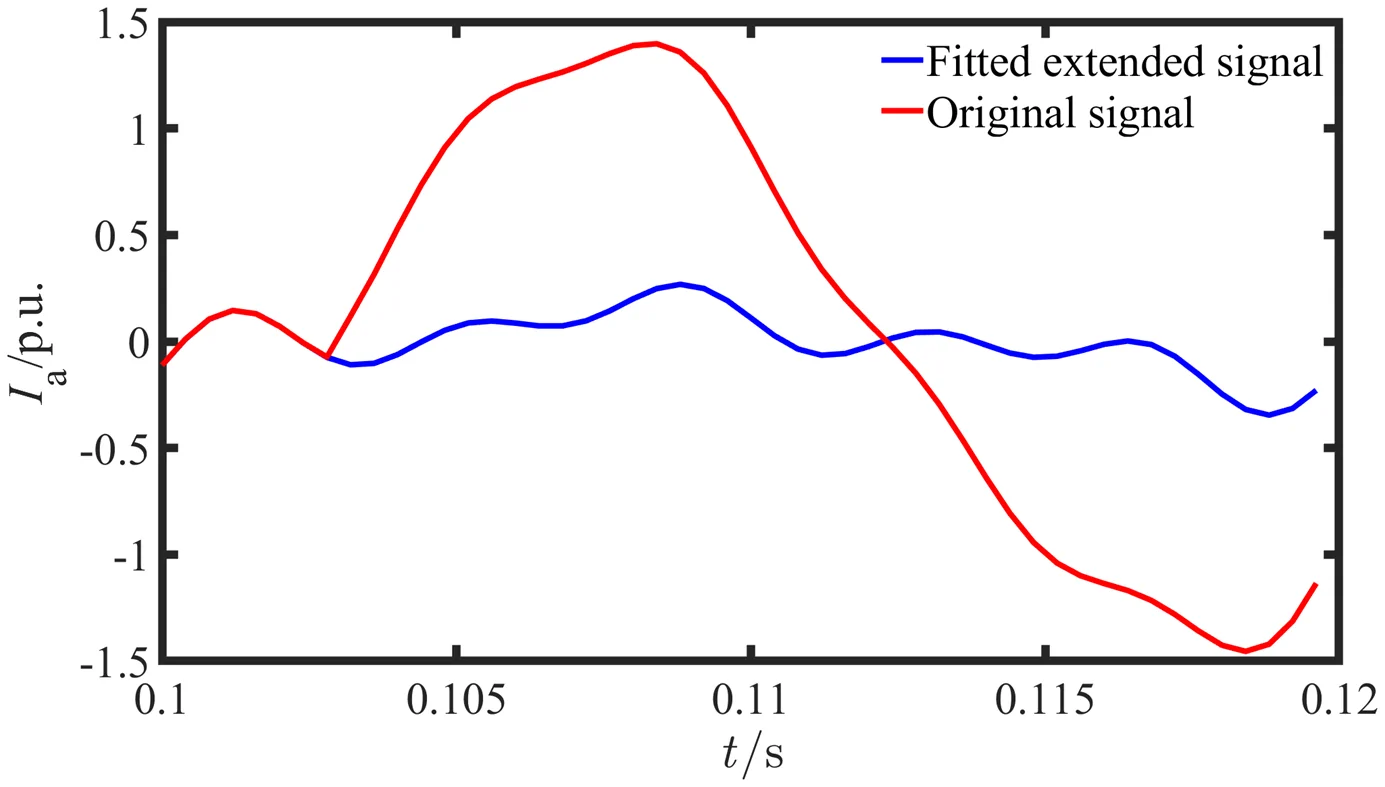
Raw data for pixel column fault detection of the fault signal containing harmonics and attenuation factors.

**Fig 9:**
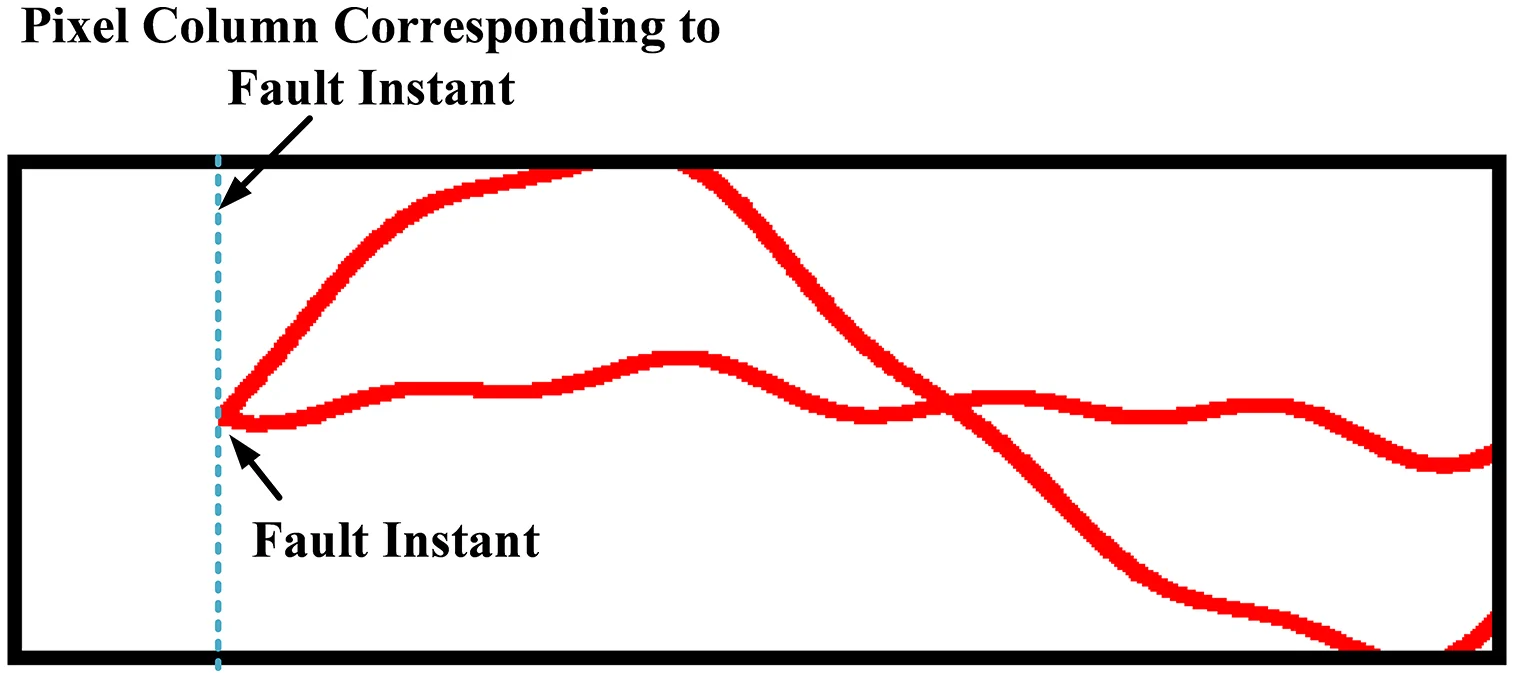
Comparative image recognition for pixel column fault detection of the fault signal containing harmonics and attenuation factors.

This section introduces a Gaussian filtering algorithm to perform convolutional smoothing on signal images, effectively suppressing fitting spikes and scattered noise. This process purifies the difference image, providing a clear and reliable visual foundation for the subsequent precise localization of the fault occurrence time.

## 4. Fault time determination

Based on the pixel-level fault localization established previously, the specific columns containing fault signatures have been identified. To determine the fault time, it is necessary to correlate each column of pixels with time information, thereby accurately obtaining the time corresponding to the fault occurrence moment. The timeline is evenly distributed across each column of the pixel image, ensuring that each column of pixels represents a specific time point or time period. In this way, when the algorithm detects the pixel column where the fault occurs, the exact time of the fault can be directly determined based on the time information corresponding to that column. For a single segment, the fault time calculation formula can be expressed as:


$$\begin{array}{c} \left\{ \begin{array}{c} @l@T_{\text{s}}=\frac{t\left(n_{\text{s}}\right)-t\left(1\right)}{n_{\text{s}}}\\ T_{\text{i}}=T_{\text{s}}\cdot (i-1)+t\left(1\right),i\in \left[1,n_{\text{s}}\right] \end{array}\right. \end{array}$$ (14)

where $t\left(n_{\text{s}}\right)$ and t(1) are the time information of the last and the first sampling points within the original sampled data segment.$n_{\text{s}}$ is the number of pixel columns in each segment; $T_{\text{s}}$ is the time difference between two pixel columns; $T_{\text{i}}$ is the time information of the i-th pixel column. Based on the above analysis, the flow of the fault time detection algorithm proposed in this paper is shown in Fig 10.

**Fig 10:**
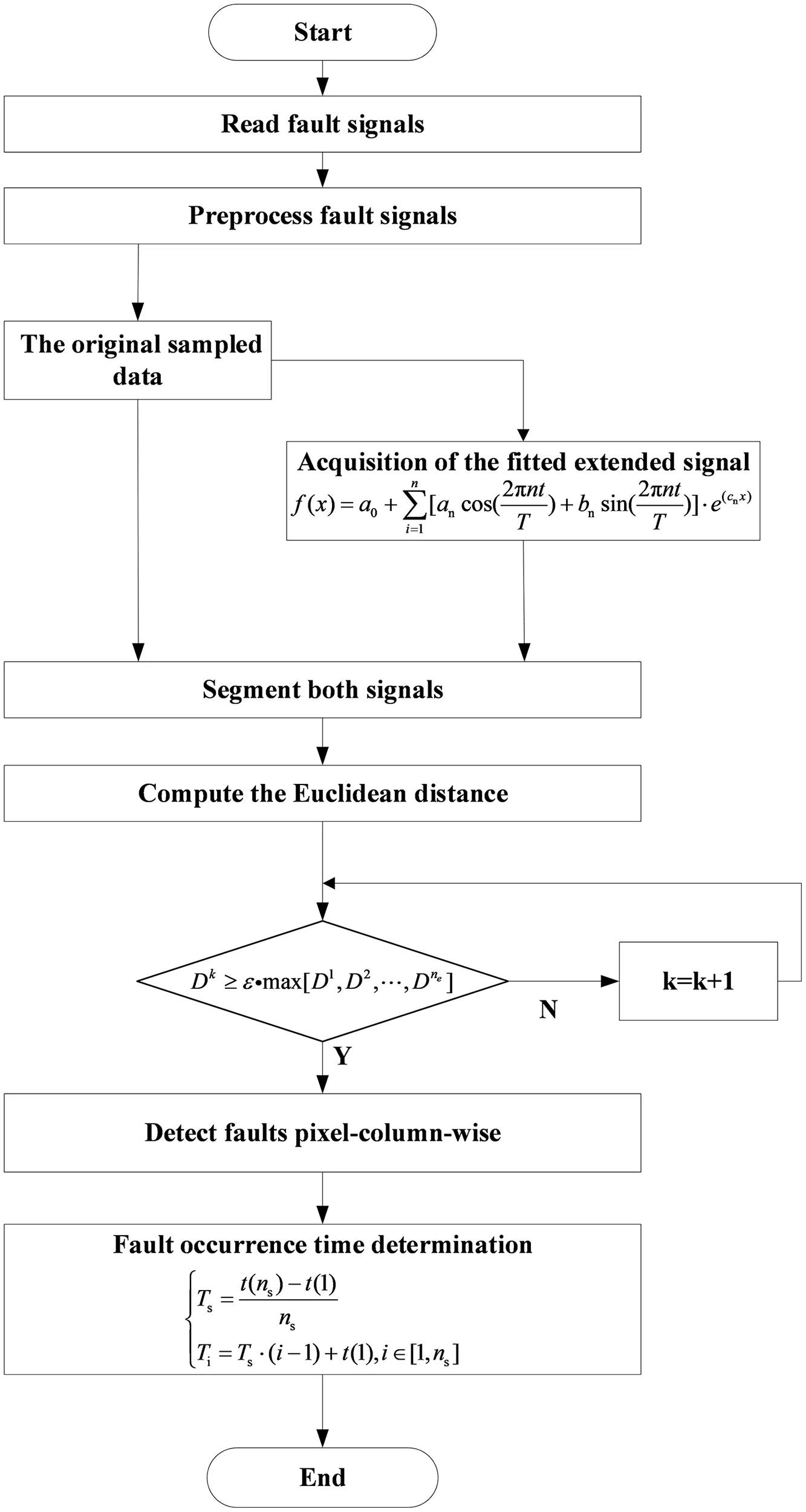
Algorithm flow of fault time detection.

## 5. Simulation analysis

This study employs MATLAB/Simulink to develop a system simulation model, which is subsequently utilized to validate the proposed fault-time accurate identification method. The simplified simulation structure diagram is shown in Fig 11. The simulation model includes five feeders with lengths of 15 km, 10 km, 18 km, 9 km, and 12 km, respectively. Other parameters are set as follows: the system power supply voltage is 110 kV, the frequency is 50 Hz, the system integrates a single distributed generator, which is modeled as a wind turbine, and the inductance value *L*_g_ of the arc suppression coil is 0.5711 H. The fault starts at 0.103 s. The first feeder is divided into segments of 1 km and 14 km, whereas the fifth feeder is divided into segments of 5 km and 7 km. The first and second feeders are overhead lines, the third and fourth feeders are cable lines, and for the fifth feeder, the segment close to the busbar is a cable line, whereas the segment far from the busbar is an overhead line. The line parameters are shown in Table 1. The developed power system model is capable of simulating faults under various complex operating conditions, thereby comprehensively validating the practicality and accuracy of the proposed fault-time identification method.

**Table 1:** Electrical parameters of power transmission lines.

Types of Power Lines	Phase Sequence	R/(Ω/km)	L/(mH/km)	C/(uF/km)
Overhead Lines	Positive Sequence	0.0137	1.21	0.01
	Zero Sequence	0.2300	5.48	0.006
Cable Lines	Positive Sequence	0.2650	2.55	0.170
	Zero Sequence	2.5400	1.02	0.153

**Fig 11:**
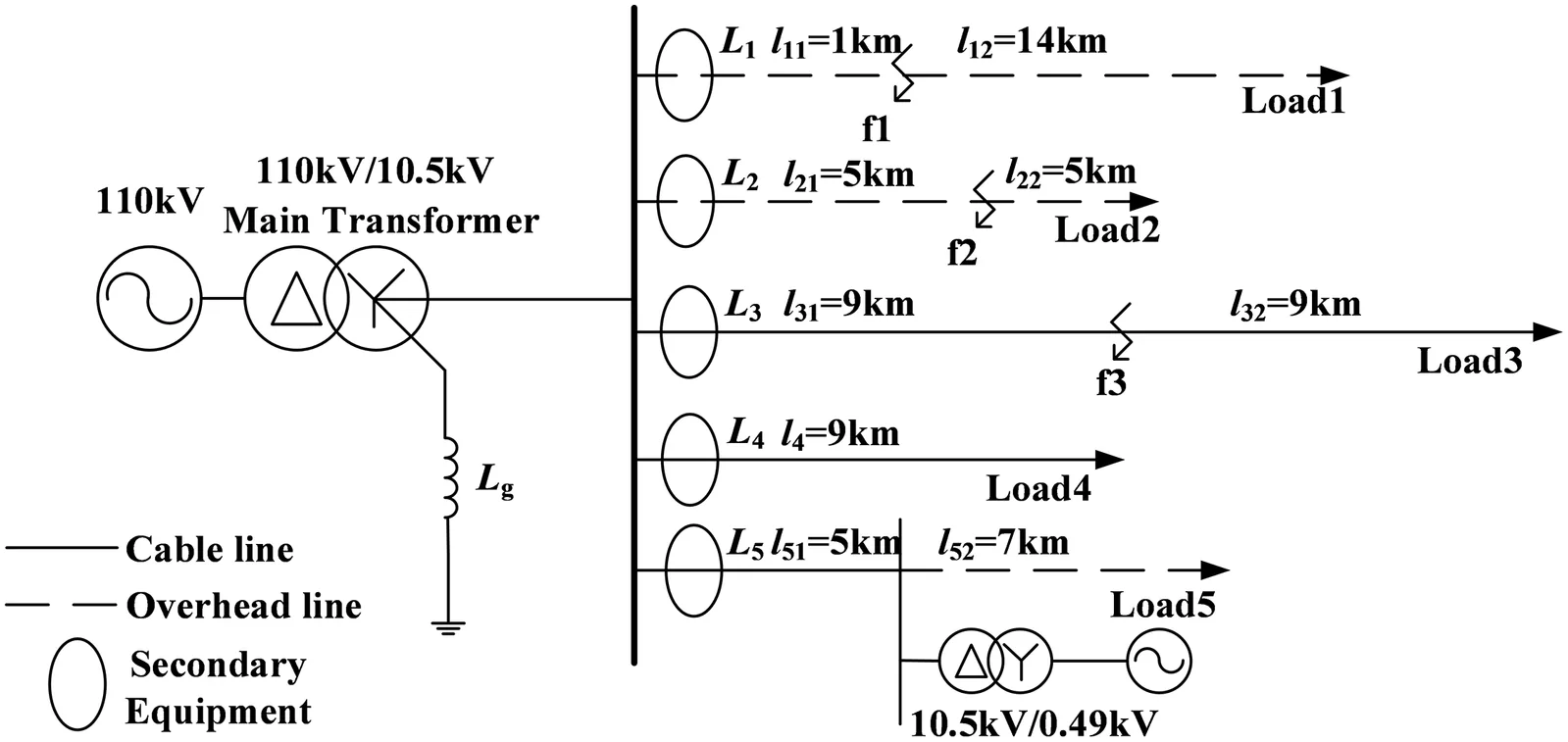
Model of 10 kV active distribution network.

### 5.1 Adaptive response to harmonic frequency components

Harmonic distortions can distort the waveform of fault signals, thereby interfering with accurate identification of the fault inception time. To verify the adaptability of the proposed algorithm to harmonics, an AB two-phase short-circuit fault with a transition resistance of 10 Ω was set at *f*_1_, and the sampling rate was set to 2500 Hz. Harmonics with different amplitudes were added to the system shown in Fig 11 for validation. The detection results are presented in Table 2.

**Table 2:** Detection errors at fault time under different harmonic amplitudes.

Harmonic Order	Amplitude	Actual Fault Inception Instant(s)	Detected Fault Instant(s)	Detection Error(ms)
3	0.10	0.103	0.102915	0.085
5	0.10	0.103	0.102915	0.085
7	0.10	0.103	0.102915	0.085
3	0.15	0.103	0.102915	0.085
5	0.15	0.103	0.102915	0.085
7	0.15	0.103	0.102915	0.085
3	0.20	0.103	0.102915	0.085
5	0.20	0.103	0.102915	0.085
7	0.20	0.103	0.102915	0.085
3	0.25	0.103	0.102915	0.085
5	0.25	0.103	0.102915	0.085
7	0.25	0.103	0.102915	0.085

From the data in Table 2, it can be observed that as the injected harmonic amplitude gradually increases from 10% to 25% of the rated current, the error in fault detection timing using the proposed algorithm remains consistently within 0.1 ms. Even with simultaneous increases in both harmonic amplitude and frequency, the error remains largely unchanged, demonstrating the high accuracy of the proposed high-order Fourier algorithm in fitting higher-order harmonics. The method thus maintains reliable performance in diverse harmonic conditions, demonstrating strong tolerance for harmonic distortion.

### 5.2 Dynamic response capability to attenuating oscillatory phenomena

Damped oscillatory components can mask the abrupt changes in signals caused by faults, increasing the difficulty of precisely capturing the initial fault moment. To verify the adaptability of the proposed algorithm to damped oscillations, an AB two-phase short-circuit fault with a transition resistance of 10 Ω was set at *f*_1_, with a sampling rate set to 2500 Hz. Damped oscillatory signals with varying amplitudes and damping factors at 50 Hz were injected into the system shown in Fig 11 for validation. The detection results are presented in Table 3.

**Table 3:** Detection errors at fault time under different damped oscillations.

Amplitude	Attenuation Constant	Actual Fault Inception Instant(s)	Detected Fault Instant(s)	Detection Error(ms)
0.10	−1	0.103	0.102915	0.085
0.10	−3	0.103	0.102953	0.047
0.10	−6	0.103	0.102990	0.010
0.15	−1	0.103	0.102915	0.085
0.15	−3	0.103	0.102953	0.047
0.15	−6	0.103	0.102990	0.010
0.20	−1	0.103	0.102915	0.085
0.20	−3	0.103	0.102953	0.047
0.20	−6	0.103	0.102990	0.010
0.25	−1	0.103	0.102915	0.085
0.25	−3	0.103	0.102953	0.047
0.25	−6	0.103	0.102990	0.010

From the data in Table 3, it can be observed that the proposed algorithm achieves a detection error of less than 0.1 ms when identifying damped oscillatory signals. The detection error remains unaffected by the amplitude of the injected signals but shows higher sensitivity to variations in the damping constant. As the damping constant increases, the signals exhibit faster decay rates, causing them to attenuate to zero more quickly. This accelerated decay reduces the influence of the superimposed oscillatory components, thereby decreasing the algorithm’s detection error and improving the accuracy of fault timing determination.

### 5.3 Robust performance under varying contact resistance conditions

The presence of transition resistance reduces both the amplitude and abruptness of fault currents, potentially leading to delayed or failed fault detection. To validate the adaptability of the proposed algorithm to different transition resistances, an AB two-phase short-circuit fault was set at *f*_1_ in the model shown in Fig 11.The transition resistance at the fault point was systematically varied, and the fault phase current waveforms were analyzed on both the M side (source side) and N side (load side) of the transmission line. The fault detection timing results are presented in Table 4.

**Table 4:** Detection errors at fault time under different transition resistors.

Transition Resistance (Ω)	Detection Location	Actual Fault Inception Instant(s)	Detected Fault Instant(s)	Detection Error(ms)
10	M	0.103	0.102953	0.047
10	N	0.103	0.102915	0.085
20	M	0.103	0.102953	0.047
20	N	0.103	0.102915	0.085
30	M	0.103	0.102953	0.047
30	N	0.103	0.102915	0.085
40	M	0.103	0.102953	0.047
40	N	0.103	0.102915	0.083
50	M	0.103	0.102953	0.047
50	N	0.103	0.102915	0.085

The interphase short-circuit transition resistance generally does not exceed 50 Ω. As can be seen from Table 4, even with a transition resistance of 50 Ω, the fault timing detection error of the proposed algorithm remains below 0.1ms. The detection errors on both the M side and N side are nearly identical. These results demonstrate the algorithm’s excellent adaptability to variations in transition resistance.

### 5.4 Multi-category short-circuit condition adaptability and robustness

Different types of short-circuit faults manifest in distinct current signatures, posing a challenge to the universal applicability of detection algorithms. To verify the adaptability of the algorithm proposed in this paper to different types of short-circuit faults, a single-phase ground fault, two-phase short-circuit, two-phase short-circuit grounding, and three-phase short-circuit with a transition resistance of 10 Ω are respectively set at *f*_1_ of the model shown in Fig 11. The fault phase current waveforms were analyzed on both the M side and N side of the transmission line. The fault detection timing results are presented in Table 5.

**Table 5:** Detection errors of fault time under different short-circuit fault types.

Short Circuit Fault Types	Detection Location	Actual Fault Inception Instant(s)	Detected Fault Instant(s)	Detection Error(ms)
single-phase grounding	M	0.103	0.102915	0.085
	N	0.103	0.102953	0.047
two-phase short-circuit	M	0.103	0.102953	0.047
	N	0.103	0.102915	0.085
two-phase short-circuit grounding	M	0.103	0.102915	0.085
	N	0.103	0.102915	0.085
three-phase short-circuit	M	0.103	0.102953	0.047
	N	0.103	0.102990	0.010

From Table 5, it can be observed that the proposed algorithm can accurately identify the fault occurrence time for various short-circuit fault types, with a detection error of less than 0.1 ms, demonstrating its strong applicability across different fault types.

### 5.5 Variable sampling frequency compatibility and robust performance

The sampling rate directly determines the temporal resolution of the signal. An insufficiently low sampling rate may lead to loss of critical signal details, thereby increasing the detection error in determining the exact fault timing. To verify the adaptability of the algorithm proposed in this paper to different sampling rates, an AB two-phase short-circuit fault was simulated at location *f*_1_ in the model shown in Fig 11 withsampling rates of 2000 Hz, 2500 Hz, and 4000 Hz respectively. The fault phase current waveform data from both the M side and N side of the fault location were analyzed, and the fault instant detection results are presented in Table 6.

**Table 6:** Detection errors at different sampling rates.

Sampling Rate(Hz)	Detection Location	Actual Fault Inception Instant(s)	Detected Fault Instant(s)	Detection Error(ms)
2000	M	0.103	0.103102	0.102
	N	0.103	0.103140	0.140
2500	M	0.103	0.102953	0.047
	N	0.103	0.102915	0.085
4000	M	0.103	0.103028	0.028
	N	0.103	0.103065	0.065

As shown in Table 6, at the lower sampling rate of 2000 Hz, the fault detection time error of the proposed algorithm exceeds 0.1 ms. This is because a reduced sampling rate decreases the number of available signal sampling points, limiting the reference data for the high-order Fourier algorithm and reducing its fitting accuracy, which in turn increases the error.

With higher sampling rates, the detection error gradually diminishes. Increasing the sampling rate at lower ranges, such as from 2000 Hz, leads to a significant reduction in error. However, once the sampling rate reaches 2500 Hz, further increases have only a marginal effect on improving detection accuracy. The proposed algorithm demonstrates strong adaptability even at low sampling rates such as 2000 Hz, maintaining relatively small detection errors.

### 5.6 Comprehensive fault location robustness and spatial adaptability

The amplitude and waveform characteristics of fault signals observed at measurement points vary with the fault location, which may compromise detection consistency. To verify the algorithm’s adaptability to different fault locations, AB two-phase short-circuit faults with a transition resistance of 10 Ω were simulated at locations *f*_1_, *f*_2_, and *f*_3_ respectively. With the sampling rate fixed at 2500 Hz, the fault phase current waveforms from both the M side and N side of each fault location were analyzed. The corresponding fault detection timing results are presented in Table 7.

**Table 7:** Detection error of fault time under different fault locations.

Short Circuit Fault Location	Detection Location	Actual Fault Inception Instant(s)	Detected Fault Instant(s)	Detection Error(ms)
f1	M	0.103	0.102953	0.047
	N	0.103	0.102915	0.085
f2	M	0.103	0.102953	0.047
	N	0.103	0.102915	0.085
f3	M	0.103	0.102953	0.047
	N	0.103	0.102915	0.085

As evidenced by Table 7, the proposed algorithm demonstrates precise fault instant identification across various fault locations, with detection errors consistently maintained below 0.1 ms. These results confirm the algorithm’s high detection accuracy regardless of fault location.

Simulation results demonstrate that the proposed method effectively and accurately identifies fault occurrence times under complex and variable operating conditions typical of active distribution networks. These conditions include the presence of higher-order harmonics, damped oscillatory components, varying transition resistances, multiple short-circuit fault types, different sampling rates, and diverse fault locations. The method achieves high detection precision, with errors consistently confined to an ultra-low range (generally within 0.1 ms). It also exhibits strong adaptability, robustness, and practical engineering value, offering a reliable and powerful technical foundation for improving protection reliability and fault diagnosis in active distribution networks.

### 5.7 Comparative analysis with existing techniques

To clearly demonstrate the comprehensive advantages of the method proposed in this paper over existing techniques, a systematic comparison between the fault moment detection method based on Euclidean distance and Gaussian filtering and five mainstream techniques is summarized in the table below. The comparative framework encompasses four critical performance metrics: detection accuracy, computational complexity, system scalability, and engineering applicability. This multidimensional analysis not only highlights the technical superiority of our approach but also reveals its practical implementation benefits in real-world power system environments. The selected benchmark methods represent state-of-the-art solutions in current research, including PMU-based matrix analysis, spectral radius detection, Prony algorithm fitting, morphological gradient techniques, and SVD-based approaches, thereby ensuring a comprehensive and authoritative evaluation.

As clearly illustrated in Table 8, the proposed method demonstrates superior comprehensive performance across the four key dimensions of detection accuracy, computational complexity, scalability, and engineering applicability. In terms of detection accuracy, the method achieves high-fidelity fitting of complex fault signals containing harmonics and damped oscillations through an improved high-order Fourier algorithm with an attenuation factor. Combined with the hybrid image strategy integrating Euclidean distance and Gaussian filtering, it consistently confines the detection error within 0.1 ms, overcoming the excessive sensitivity of existing methods to sampling rate, data window length, or historical data. Regarding computational efficiency, although the method involves signal fitting and image processing, the efficient implementation of Gaussian filtering and vector-based distance calculation avoids the time-consuming iterations and matrix decomptions required by methods like Prony and SVD, achieving an excellent balance between accuracy and real-time performance. In system adaptability, the method employs a fixed data window segmented by the signal period, demonstrating strong robustness across different network topologies and fault locations, without needing careful parameter adjustments like the sliding window method [24]. Finally, regarding practical application, the most significant advantage of this solution is its outstanding cost-effectiveness. It does not rely on expensive additional hardware such as PMUs or high-precision GPS modules and operates reliably using conventional instrument transformers at standard sampling rates (≥2000 Hz). This provides a highly practical technical pathway for implementing high-precision fault diagnosis within existing distribution network infrastructure.

**Table 8:** Comparative analysis of different fault detection methods.

Performance Dimensions	Proposed Method	Prony Algorithm-based Fitting [27]	Singular Value Decomposition (SVD)-based Approaches [26]
Detection Accuracy	< 0.1 ms, maintaining high precision under various complex conditions	Good detection performance, but accuracy is affected by data window length and sampling frequency.	Hankel matrix columns require manual selection, lacking adaptability.
Computational Complexity	Moderate, strikes a balance between accuracy and computational efficiency.	High, requires iterative parameter tuning and matrix operations, computationally intensive.	High, involves matrix construction and decomposition, computationally heavy.
Scalability & Adaptability	Strong, uses a fixed-length data window segmented by signal period for consistent performance.	Sensitive to data window length, limiting its adaptability.	Matrix structure requires manual setup, lacks adaptability.
Engineering Applicability & Cost	High, highly compatible with existing infrastructure; cost-effective.	Primarily algorithm-dependent, but high computational resource demands may impact real-time performance.	May require specialized sensors or high computing resources, increasing deployment costs.

To further visually demonstrate the differences in detection performance, Fig 12 presents the fault detection results obtained by the method of reference [27] under the same test conditions (AB two-phase short-circuit fault, transition resistance 10 Ω, fault inception time 0.103 s, sampling rate 2500 Hz).

**Fig 12:**
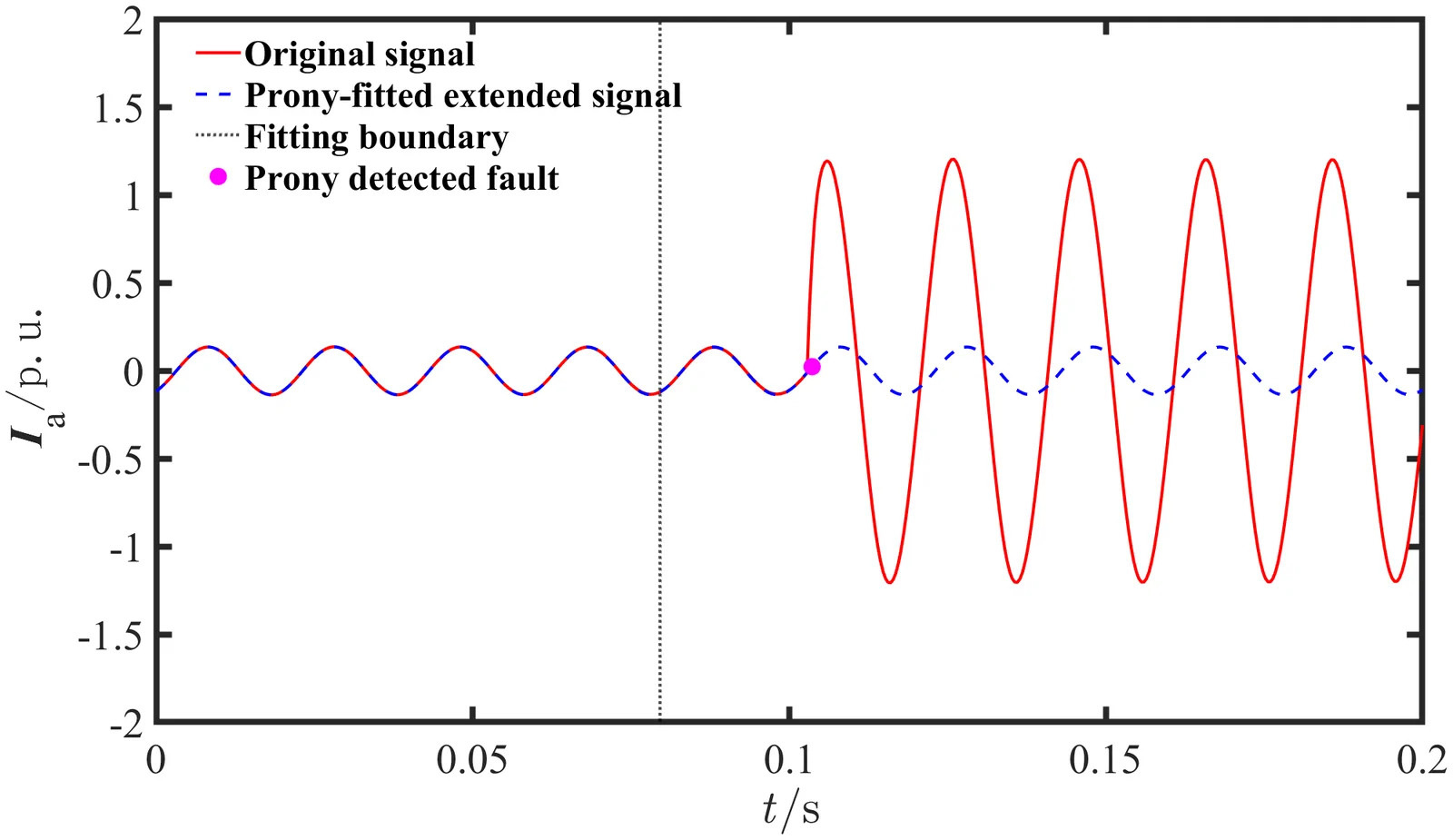
Fault detection result using Prony fitting.

From Fig 12, it can be observed that there is a noticeable deviation between the Prony-fitted signal and the original signal near the fault inception time (0.103 s). Specifically, the fault time detected by the Prony-based method is slightly delayed compared to the actual fault inception instant. This fitting deviation near the fault point is subsequently propagated to the pixel-column comparison stage, thereby adversely affecting the accuracy of the subsequent pixel-difference detection. The raw data used to generate Fig 12 is provided in S3 Data.

Fig 13 presents the fault detection results obtained by the method in reference [27] based on pixel-column comparison. In this figure, the horizontal axis represents the image pixel columns (corresponding to the time axis), and the vertical axis represents the pixel difference values, reflecting the per-pixel difference distribution between the original signal image and the Prony-fitted signal image. Before fault occurrence, the pixel difference values remain at a relatively low level. At the pixel column corresponding to the fault inception instant, a significant jump in the difference value can be observed. However, due to the inherent fitting errors of the Prony algorithm, the pixel differences in Segment 5 are abnormally elevated, which leads to an incorrect identification of the fault segment.

**Fig 13:**
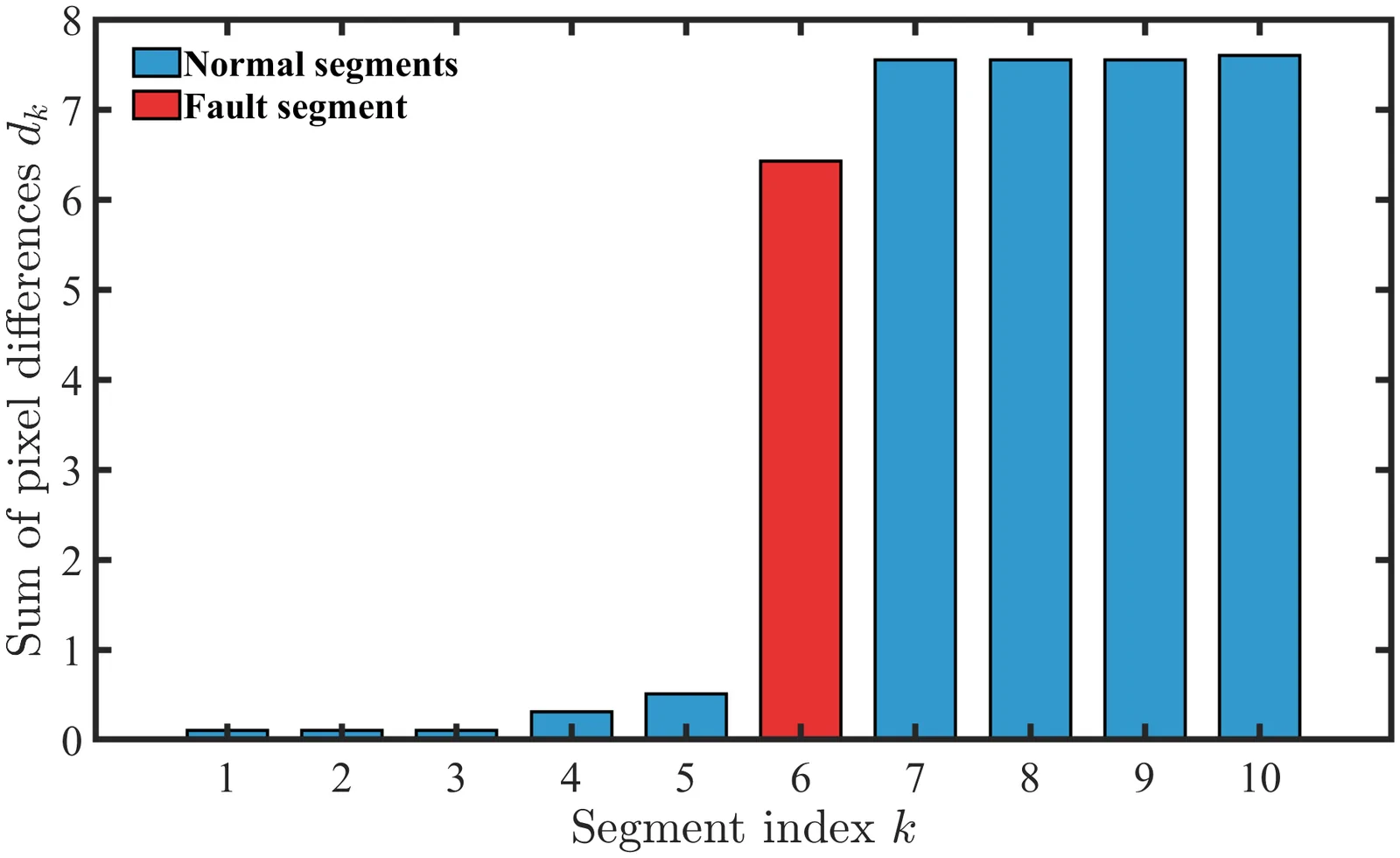
Pixel-column difference detection diagram using the method in reference [27].

Fig 14 presents the final fault inception time detection result obtained by the method in reference [27]. In this figure, the detected fault inception instant is marked on the original signal waveform using specific markers (such as vertical lines or arrows). It can be observed that the detected fault time is noticeably delayed relative to the actual fault inception time (0.103 s), with an error of approximately 0.2 ms. This delay is primarily attributed to the fitting deviation of the Prony algorithm near the fault point, which propagates to the pixel-column comparison stage and consequently shifts the localization of the fault-initiating pixel column. This figure intuitively reflects the insufficiency of the method in reference [27] regarding the accuracy of fault timing detection.

**Fig 14:**
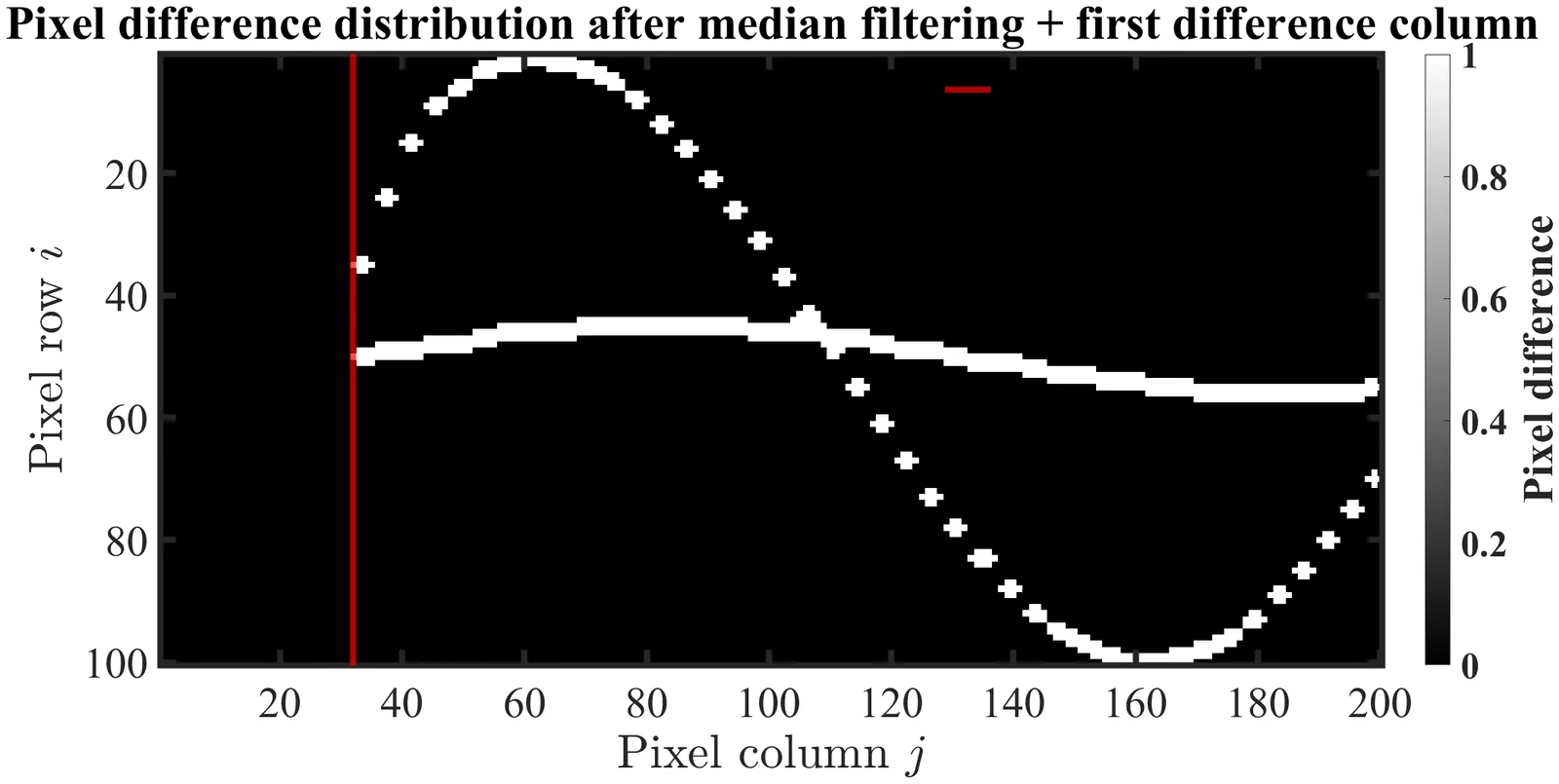
Pixel-column fault inception time detection result using the method in reference [27].

In summary, Section 5.7 systematically presents the complete detection process of the method in reference [27] through Fig 12, Fig 13, and Fig 14, which sequentially illustrate signal fitting, pixel-difference detection, and fault inception time localization. This step-by-step exposition reveals the detection delay issue caused by the insufficient fitting accuracy of the Prony algorithm. On this basis, combined with the multi-dimensional performance comparison in Table 8, it is fully demonstrated that the proposed method based on Euclidean distance and Gaussian filtering exhibits significant advantages in detection accuracy and anti-interference performance. Under baseline, damped oscillation, and low-sampling-rate conditions, the proposed method consistently achieves the best detection accuracy and robustness among all compared methods, validating its effectiveness and reliability for practical engineering applications.

## 6. Field data validation

Simulation results demonstrate that the proposed method achieves high accuracy in identifying the inception time of faults at various locations. To further validate the method, field measurement data were employed. The field measurement data used in this study were collected from two substations: Xi’an Substation (Shaanxi Province, China) and Jiangsu Substation (Jiangsu Province, China). Access to the field sites and the use of the recorded fault waveform data for research purposes were officially approved by the Shaanxi Provincial Electric Power Company (State Grid Shaanxi Electric Power Company) and the Jiangsu Provincial Electric Power Company (State Grid Jiangsu Electric Power Company). No additional field access permit was required because the data were obtained from routine operational recordings rather than from active interventions or access to restricted areas involving endangered species or protected habitats. The first validation case employs the Phase A voltage values during a Phase B-to-ground fault, with a fault inception time of 0.075 s, a sampling rate of 9600 Hz, and 1920 sampling points. Fig 15 presents a comparison between the raw signals of phase A voltage and current waveforms collected on-site and their fitted extended signals. The raw data used to generate Fig 15 is provided in S4 Data.

**Fig 15:**
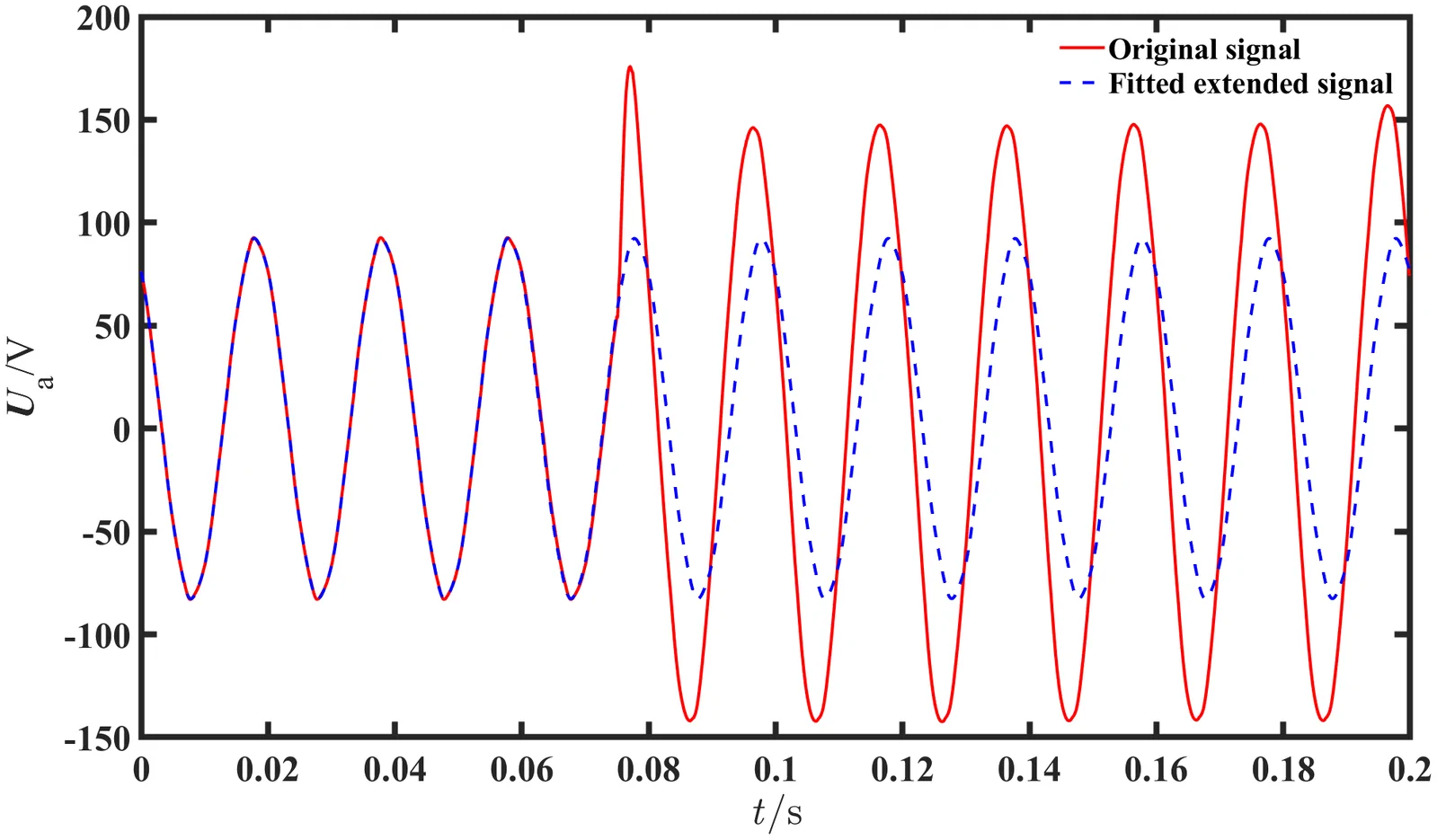
Comparison of the original and fitted extended signals for the phase A voltage waveform.

An analysis was performed on the original signal at intervals of 0.02 s. The analysis of the computed Euclidean distances for each segment (Fig 16) revealed that the value for segment 4 was a significant outlier. According to the criterion established in Formula (9), this segment was thus identified as faulty. Figs 17 and 18 provide a pixel-based comparison between the original and fitted extended signals for the segment containing the fault occurrence moment. For segment 4 of the original fault voltage data, the corresponding image has a pixel width of 485 columns. The first detected fault occurrence is at the 347th pixel column, corresponding to an actual time of 0.0743 s. Fig 18 demonstrates that the proposed algorithm can accurately identify the fault occurrence time with a detection error of less than 0.1 ms, which is consistent with the simulation results.

**Fig 16:**
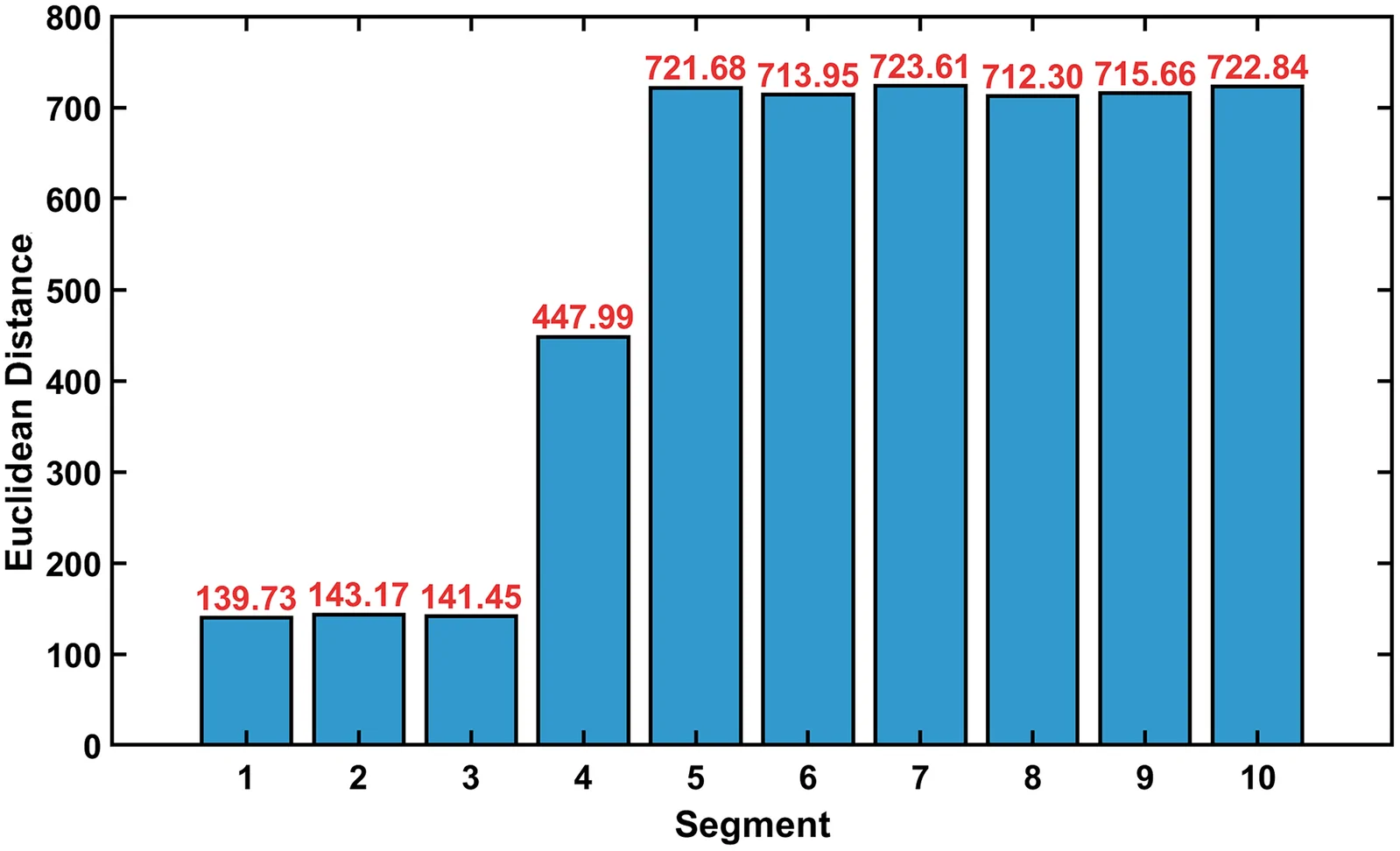
Euclidean distances of each segment for the phase A voltage waveform.

**Fig 17:**
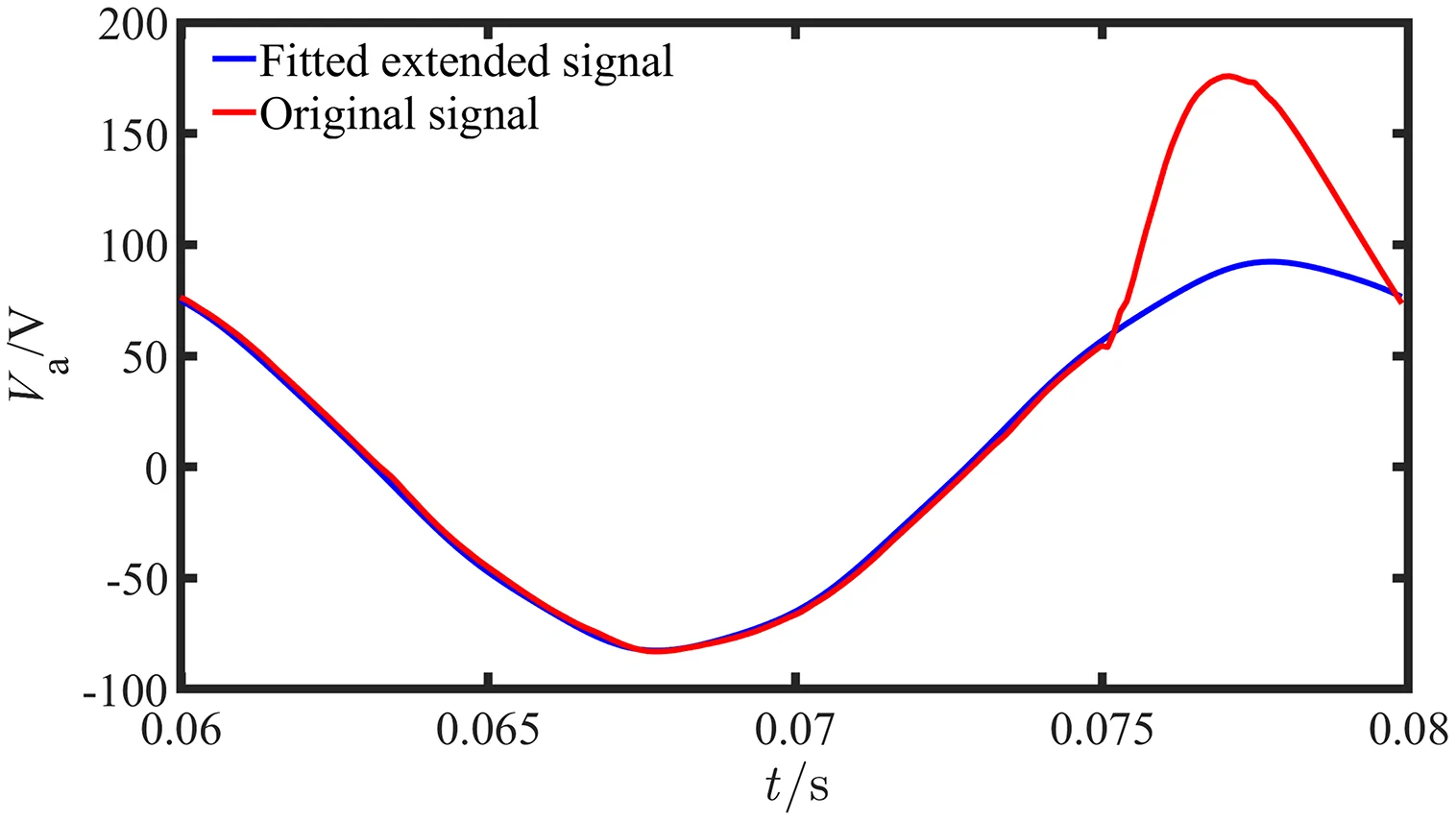
Raw data for pixel column fault detection of the phase A voltage waveform.

**Fig 18:**
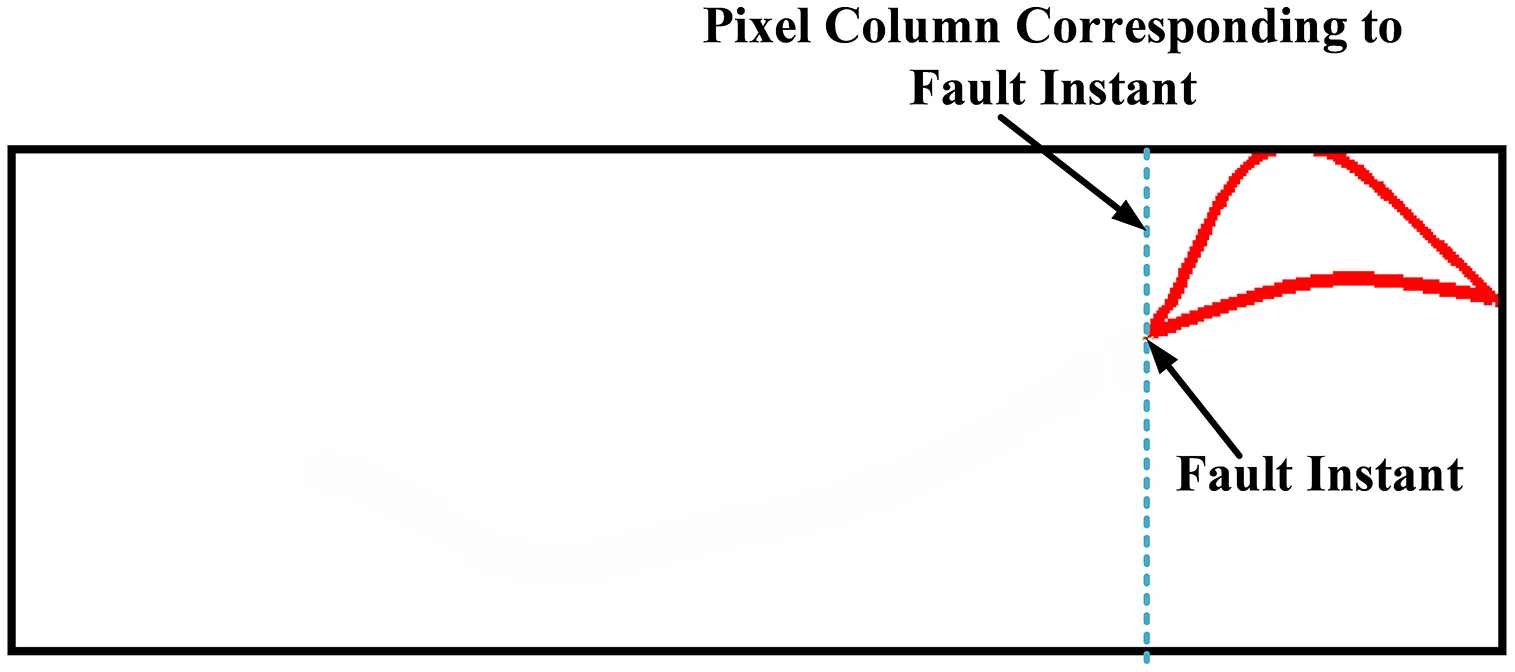
Comparative image recognition for pixel column fault detection of the phase A voltage waveform.

The second scenario involves an over-resistance fault between phase A and phase B on the high-voltage side within the protected zone. The fault inception time is 0.098 s, with a sampling rate of 2000 Hz comprising 400 sampling points. The fault identification is performed using the current waveform signal from phase A. Fig 19 presents a comparison between the raw signals of phase A voltage and current waveforms collected on-site and their fitted extended signals.

**Fig 19:**
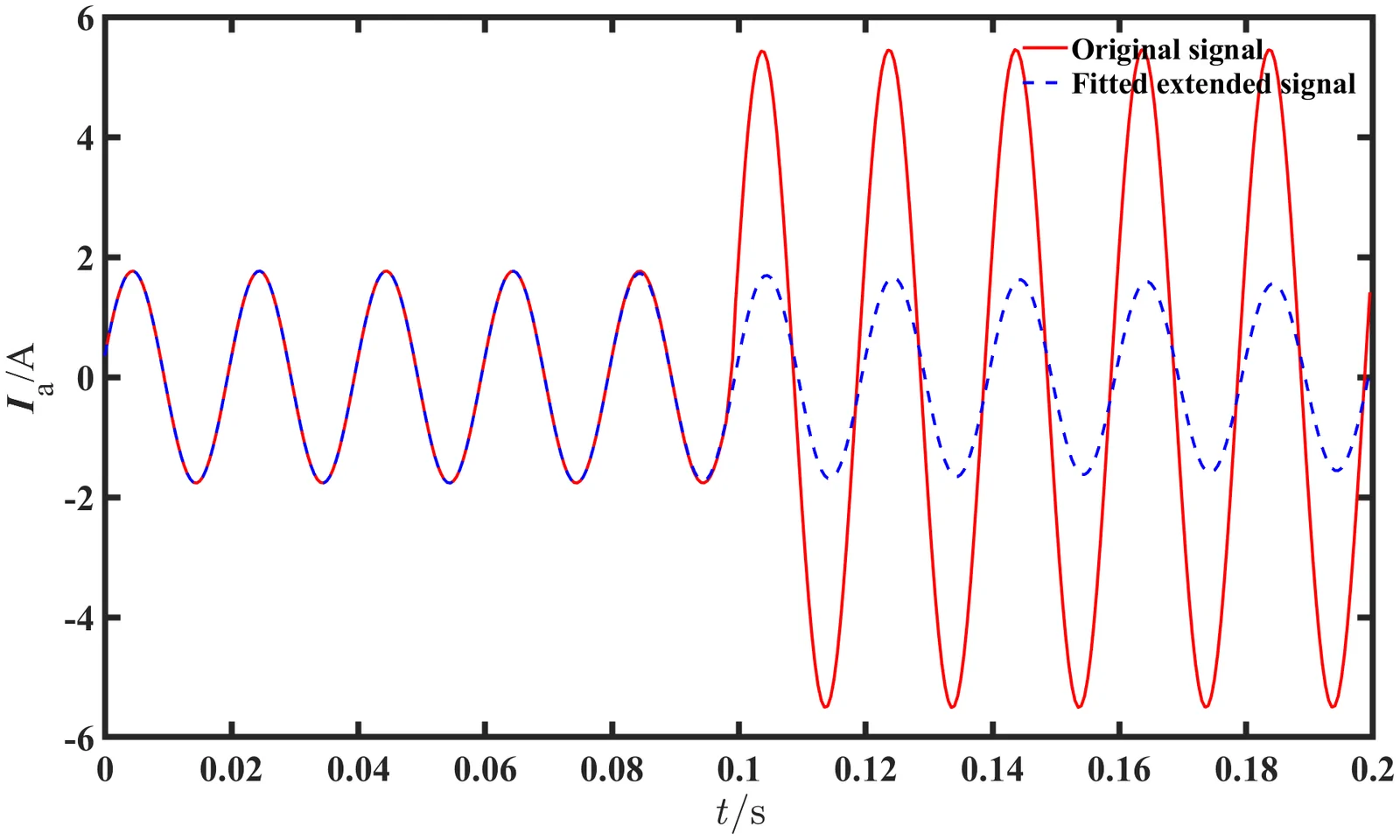
Comparison of the original and fitted extended signals for the phase A current waveform.

Fig 20 shows the calculated Euclidean distances for different segments, from which it is clearly visible that the fault initiates from segment 5. Figs 21 and 22 provide a pixel-based comparison between the original and fitted extended signals for the segment containing the fault occurrence moment. The first detected fault occurrence was at the 430th pixel column, corresponding to an actual time of 0.09773 s. The results in Fig 22 demonstrate that the proposed algorithm can accurately identify the fault occurrence time with a detection error of less than 0.1 ms, which is consistent with the simulation results.

**Fig 20:**
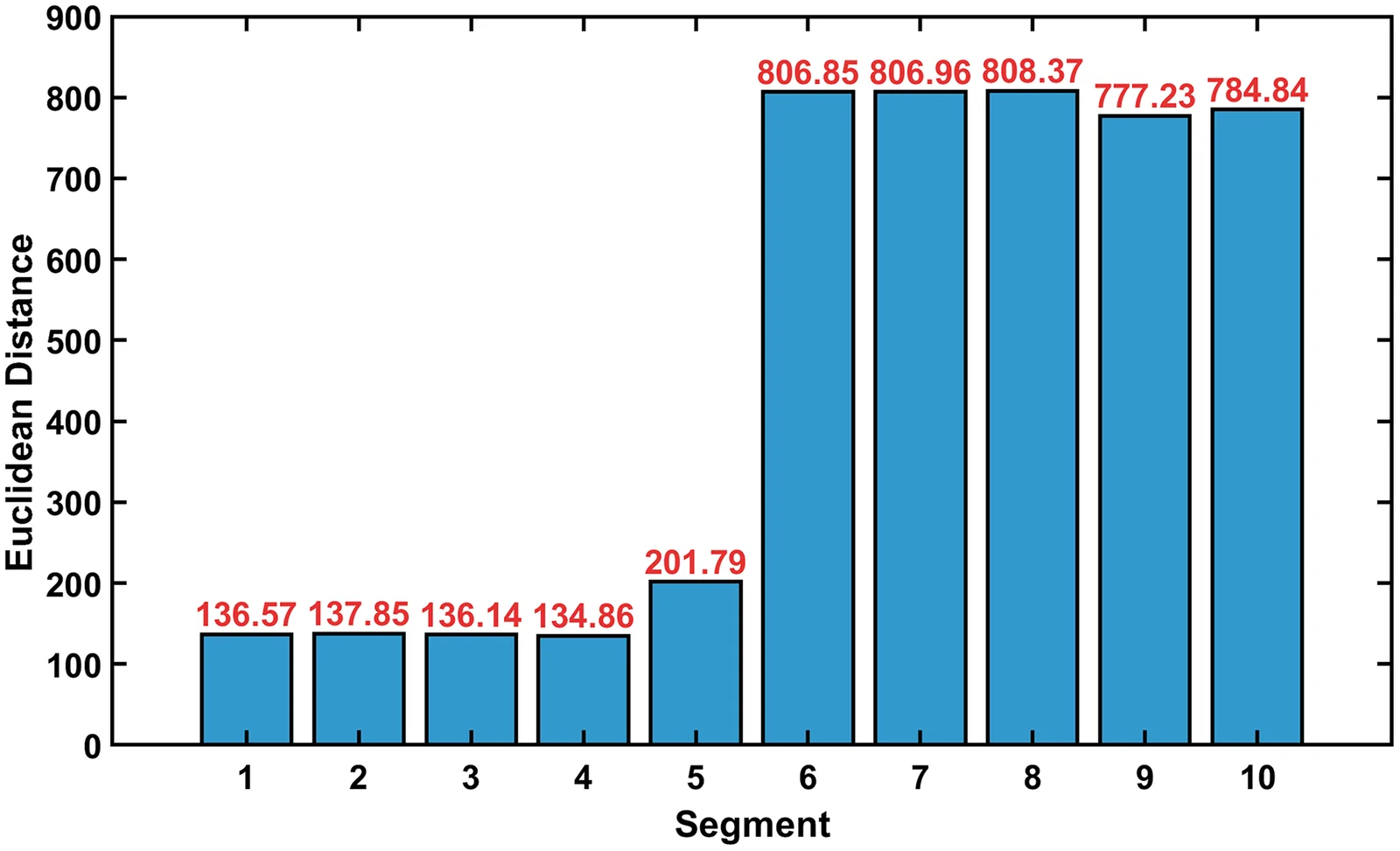
Euclidean distances of each segment for the phase A current waveform.

**Fig 21:**
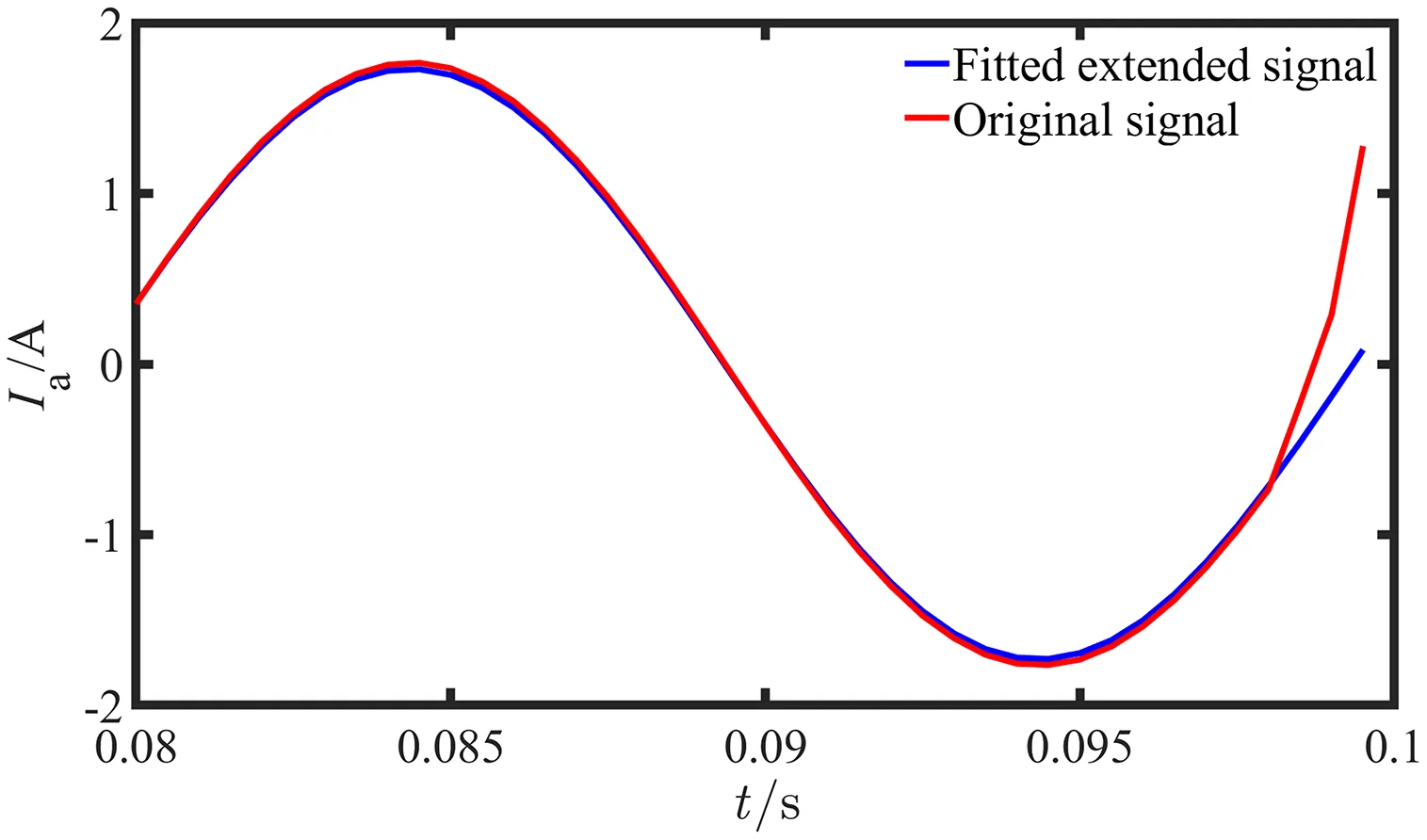
Raw data for pixel column fault detection of the phase A current waveform.

**Fig 22:**
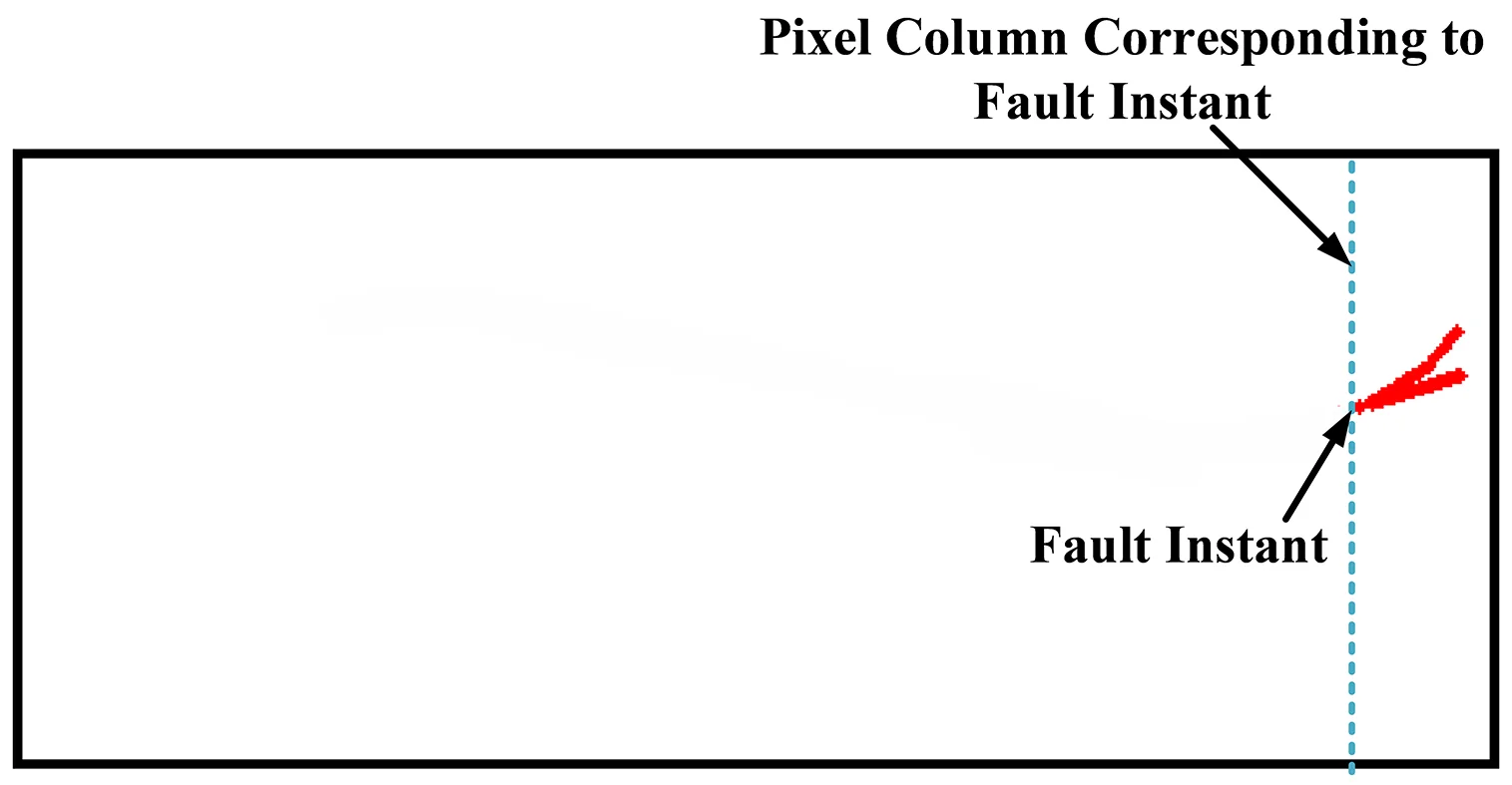
Comparative image recognition for pixel column fault detection of the phase A current waveform.

The proposed method demonstrates excellent performance in both simulation and field data validation, as it is capable of accurately identifying fault occurrence times under various complex conditions with detection errors generally below 0.1 ms. Combining high precision and strong robustness, the method is applicable to different harmonics, oscillations, transition resistances, and fault types, while remaining compatible with conventional sampling equipment. With its favorable engineering applicability and cost-effectiveness, it provides a reliable and practical technical solution for real-time fault diagnosis in active distribution networks.

## 7. Conclusion

As active distribution networks continue to evolve, their increasingly complex operating environments have exposed the limitations of conventional fault detection methods in both efficiency and accuracy. In response, This paper has presented a fault time detection method for active distribution networks that, for the first time, combines three established techniques—damping-adaptive high-order Fourier fitting, Euclidean distance-based segment localization, and Gaussian filtering-aided pixel comparison—into a coarse-to-fine architecture tailored for signals containing harmonics and damped oscillations. While each individual technique is known, their integration and application to sub-0.1 ms fault timing under low sampling rates (≥2.5 kHz) is novel. The proposed architecture addresses two fundamental limitations of prior art: the inability of Prony-based methods to handle damping variations, and the sensitivity of direct pixel comparison to fitting residuals.

To further clarify the level of novelty, a point-by-point comparison with the most relevant existing methods is provided. Compared with the Prony + pixel comparison method [27], the proposed method differs in three key aspects. First, it introduces a damping factor to achieve high-fidelity fitting of damped oscillatory signals, whereas [27] cannot handle such components. Second, it applies Gaussian filtering to suppress fitting residuals, while [27] performs direct pixel comparison without preprocessing, making it sensitive to noise. Third, it adopts a coarse-to-fine two-stage strategy that uses Euclidean distance for coarse localization before pixel-wise refinement, thereby reducing computational burden—a step that is absent in [27]. Similarly, when compared with the SVD-based method [26], the proposed method offers two distinct advantages. On one hand, it employs a fixed window segmented by the signal period instead of manually setting the number of Hankel matrix columns, eliminating the need for subjective parameter selection. On the other hand, it achieves automatic parameter tuning through an adaptive threshold criterion (Eq 6), whereas the SVD method’s performance heavily depends on manual choices.

Before discussing the performance, it is worth noting that the proposed method was developed and validated specifically for 10 kV active distribution networks, which represent a typical and widely deployed voltage level in medium-voltage distribution systems. Then, this approach employs an enhanced higher-order Fourier algorithm incorporating an attenuation factor, enabling high-fidelity reconstruction of fault signals containing higher-order harmonics and damped oscillatory components. By transforming time-domain signals into pixel-based representations and applying Gaussian filtering to eliminate fitting errors, the method achieves precise fault segment localization through Euclidean distance calculation and pixel-level analysis. The proposed technique demonstrates remarkable robustness across various challenging conditions, maintaining detection errors within 0.1 ms despite the presence of harmonics, damped oscillations, varying transition resistances, and different fault types. Furthermore, its compatibility with conventional sampling equipment and adaptability to different network topologies highlight its significant engineering applicability. Leveraging advances in distributed computing, this detection paradigm offers an optimal balance between computational efficiency and accuracy, presenting a practical and reliable solution for real-time fault diagnosis in modern power systems.

In summary, this detection method presents a practical and reliable solution for real-time fault diagnosis in 10 kV active distribution systems. Extension to other voltage levels would require additional validation, as the method’s parameters were optimized based on the 10 kV network model used in this study. Nevertheless, since the algorithmic core is voltage-level agnostic and does not rely on topology-specific parameters, the method is expected to be transferable with minor recalibration.

## Supporting information

S1 Data Raw data for Fig 1, Fig 2, and Fig 7 in the manuscript. (CSV)

S2 Data Raw data for Fig 3 in the manuscript. (CSV)

S3 Data Raw data for Fig 12 in the manuscript. (CSV)

S4 Data Raw data for Fig 15 in the manuscript. (CSV)

## References

[1] Li J, Zhou X, Zhou Y, Han A. Optimal configuration of distributed generation based on an improved beluga whale optimization. IEEE Access. 2024;12:31000–13. doi: 10.1109/access.2024.3368440

[2] Mirshekali H, Dashti R, Keshavarz A, Torabi AJ, Shaker HR. A novel fault location methodology for smart distribution networks. IEEE Trans Smart Grid. 2021;12(2):1277–88. doi: 10.1109/tsg.2020.3031400

[3] Blaabjerg F, Yang Y, Yang D, Wang X. Distributed power-generation systems and protection. Proc IEEE. 2017;105(7):1311–31. doi: 10.1109/jproc.2017.2696878

[4] Singh B, Pankaj KD. Distributed power generation planning for distribution networks using electric vehicles: Systematic attention to challenges and opportunities. J Energy Storage. 2022;48:104030.

[5] Nsaif YM, Lipu MSH, Ayob A, Yusof Y, Hussain A. Fault detection and protection schemes for distributed generation integrated to distribution network: Challenges and suggestions. IEEE Access. 2021;9:142693–717. doi: 10.1109/access.2021.3121087

[6] Rai P, Londhe ND, Raj R. Fault classification in power system distribution network integrated with distributed generators using CNN. Electric Power Syst Res. 2021;192:106914. doi: 10.1016/j.epsr.2020.106914

[7] Tavoosi J, Shirkhani M, Azizi A, Ud Din S, Mohammadzadeh A, Mobayen S. A hybrid approach for fault location in power distributed networks: Impedance-based and machine learning technique. Electric Power Syst Res. 2022;210:108073. doi: 10.1016/j.epsr.2022.108073

[8] Pignati M, Zanni L, Romano P, Cherkaoui R, Paolone M. Fault detection and faulted line identification in active distribution networks using synchrophasors-based real-time state estimation. IEEE Trans Power Delivery. 2017;32(1):381–92. doi: 10.1109/tpwrd.2016.2545923

[9] Spielböck T, Beláň A. Design of a one-sided, impedance-based transmission line fault locator using line topology and source impedances. Electric Power Syst Res. 2018;161:123–38. doi: 10.1016/j.epsr.2018.03.013

[10] Pocoma Copa EI, Quitin F, Vandendorpe L, Philippe DD, Horlin F. Self-synchronization based localization of a time-misaligned transmitter in cellular networks. IEEE Trans Veh Technol. 2022;71(4):4102–15. doi: 10.1109/tvt.2022.3148532

[11] Okumus H, Nuroglu FM. A random forest-based approach for fault location detection in distribution systems. Electr Eng. 2020;103(1):257–64. doi: 10.1007/s00202-020-01074-8

[12] Barkhi M, Pourhossein J, Hosseini SA. Integrating fault detection and classification in microgrids using supervised machine learning considering fault resistance uncertainty. Sci Rep. 2024;14(1):28466. doi: 10.1038/s41598-024-77982-7 39557975 PMC11574044

[13] Ghanizadeh Bolandi T, Yazdaninejadi A. Vulnerability assessment approach for real‐time and regional monitoring of backup protections: minimising number of GPS‐based distance relays. IET Generation Trans & Dist. 2020;14(14):2687–97. doi: 10.1049/iet-gtd.2019.1756

[14] Chavez JJ, Kumar NV, Azizi S, Guardado JL, Rueda J, Palensky P, et al. PMU-voltage drop based fault locator for transmission backup protection. Electric Power Syst Res. 2021;196:107188. doi: 10.1016/j.epsr.2021.107188

[15] Khoshbouy M, Yazdaninejadi A, Bolandi TG. Transmission line adaptive protection scheme: A new fault detection approach based on pilot superimposed impedance. Int J Electr Power Energy Syst. 2022;137:107826.

[16] Jamshidpour E, Poure P, Saadate S. Photovoltaic systems reliability improvement by real-time FPGA-based switch failure diagnosis and fault-tolerant DC–DC converter. IEEE Trans Ind Electron. 2015;62(11):7247–55. doi: 10.1109/tie.2015.2421880

[17] Bindhu V, Ranganathan G. Effective automatic fault detection in transmission lines by hybrid model of authorization and distance calculation through impedance variation. Int J Electron. 2021;3(01):36–48.

[18] Mishra SK, Tripathy LN. A critical fault detection analysis and fault time in a UPFC transmission line. Prot Control Mod Power Syst. 2019;4(1). doi: 10.1186/s41601-019-0117-5

[19] Rosero-Morillo V, Pham LNH, Salazar-Pérez S, Gonzalez-Longatt F, Orduña E. Fault detection in distribution networks with distributed generation: A practical guide to the morphological median filter for the feature extraction of faults. Eng Proc. 2024;77(1):5.

[20] Zarei SF, Khankalantary S. Protection of active distribution networks with conventional and inverter-based distributed generators. Int J Elect Power Energy Syst. 2021;129:106746. doi: 10.1016/j.ijepes.2020.106746

[21] Kazmi SAA, Shahzad MK, Khan AZ, Shin DR. Smart distribution networks: A review of modern distribution concepts from a planning perspective. Energies. 2017;10(4):501.

[22] Srivastava C, Tripathy M. Novel grounding and protection strategy for DC microgrid restraining fault current. IEEE Trans Power Delivery. 2024;39(4):2182–93. doi: 10.1109/tpwrd.2024.3397711

[23] Liu X, Ma Y, Yang D. Fault time determination and identification in power grids based on data correlation analysis. Power Syst Technol. 2022;46(4):1529–38.

[24] Dong J, Hou LG, Juan L. Fault instant detection algorithm based on curve fitting of phase current increments. Autom Electr Power Syst. 2018;38(4):75–81.

[25] Zhang YC, Gao HL, Yuan T. A fault time detection method in a distribution network based on a sudden change of current and mathematical morphology. Protect Control Mod Power. 2022;50(12):54–62.

[26] Li J, Gao H, Gong X, Kong X, Gao L. A novel fault instant detection scheme for fault data self-synchronization method in distribution network. Prot Contr Mod Pow. 2018;46(04):92–8.

[27] Yan Y, Jian W. Fault time detection method of active distribution network based on Prony fitting and pixel comparison. ICEPET. 2024;:797–802.

[28] Yao Z, Liu Y, Chen J, Ji J, Zhang M, Gong Y. Active high-impedance fault detection method for resonant grounding distribution networks. IEEE Access. 2024;12:10932–45. doi: 10.1109/access.2024.3352258

[29] Najafzadeh M, Pouladi J, Daghigh A, Beiza J, Abedinzade T. Fault detection, classification and localization along the power grid line using optimized machine learning algorithms. Int J Comput Intell Syst. 2024;17(1). doi: 10.1007/s44196-024-00434-7

[30] Li Q, Luo H, Cheng H, Deng Y, Sun W, Li W, et al. Incipient fault detection in power distribution system: A time–frequency embedded deep-learning-based approach. IEEE Trans Instrum Meas. 2023;72:1–14. doi: 10.1109/tim.2023.3250220

[31] Wahab MF, Gritti F, O’Haver TC. Discrete Fourier transform techniques for noise reduction and digital enhancement of analytical signals. TrAC Trends in Analytical Chemistry. 2021;143:116354. doi: 10.1016/j.trac.2021.116354

[32] Cardarilli GC, Di Nunzio L, Fazzolari R, Nannarelli A, Re M, Spanò S. N-dimensional approximation of euclidean distance. IEEE Transactions on Circuits and Systems II. 2019;67(3):565–9.

[33] Dong X, Peng Q, Wu H, Chang Z, Yue Y, Zeng Y. New principle for busbar protection based on the Euclidean distance algorithm. PLoS One. 2019;14(7):e0219320. doi: 10.1371/journal.pone.0219320 31339903 PMC6655625

[34] Le VL, Kim TJ, Kim YD, Aspnes DE. Extended Gaussian filtering for noise reduction in spectral analysis. J Korean Phys Soc. 2020;77(10):819–23. doi: 10.3938/jkps.77.819

[35] Fuseiller G, Marie R, Mourioux G, Duno E, Labbani-Igbida O. Enhancing distance transform computation by leveraging the discrete nature of images. J Real-Time Image Proc. 2022;19(4):763–73. doi: 10.1007/s11554-022-01221-3

